# Vagus nerve stimulation optimized cardiomyocyte phenotype, sarcomere organization and energy metabolism in infarcted heart through FoxO3A-VEGF signaling

**DOI:** 10.1038/s41419-020-03142-0

**Published:** 2020-11-12

**Authors:** Bin Luo, Yan Wu, Shu-lin Liu, Xing-yuan Li, Hong-rui Zhu, Lei Zhang, Fei Zheng, Xiao-yao Liu, Ling-yun Guo, Lu Wang, Hong-xian Song, Yan-xia Lv, Zhong-shan Cheng, Shi-you Chen, Jia-ning Wang, Jun-ming Tang

**Affiliations:** 1 Department of Physiology, Hubei Key Laboratory of Embryonic Stem Cell Research, School of Basic Medicine Science, Hubei University of Medicine, 442000 Hubei, China; 2 Institute of Biomedicine, Hubei University of Medicine, 442000 Hubei, China; 3 Institute of Clinical Medicine and Department of Cardiology, Renmin Hospital, Hubei University of Medicine, 442000 Shiyan, Hubei China; 4 Applied Bioinformatics Center, St. Jude Children’s Research Hospital, Memphis, TN USA; 5 The Department of Surgery, University of Missouri, Columbia, MO USA

**Keywords:** Cardiac hypertrophy, Heart failure

## Abstract

Vagus nerve stimulation (VNS) restores autonomic balance, suppresses inflammation action and minimizes cardiomyocyte injury. However, little knowledge is known about the VNS’ role in cardiomyocyte phenotype, sarcomere organization, and energy metabolism of infarcted hearts. VNS in vivo and acetylcholine (ACh) in vitro optimized the levels of *α/β-MHC* and *α-Actinin* positive sarcomere organization in cardiomyocytes while reducing F-actin assembly of cardiomyocytes. Consistently, ACh improved glucose uptake while decreasing lipid deposition in myocytes, correlating both with the increase of Glut4 and CPT1α and the decrease of PDK4 in infarcted hearts in vivo and myocytes in vitro, attributing to improvement in both glycolysis by VEGF-A and lipid uptake by VEGF-B in response to Ach. This led to increased ATP levels accompanied by the repaired mitochondrial function and the decreased oxygen consumption. Functionally, VNS improved the left ventricular performance. In contrast, ACh-m/nAChR inhibitor or knockdown of *VEGF-A/B* by shRNA powerfully abrogated these effects mediated by VNS. On mechanism, ACh decreased the levels of nuclear translocation of *FoxO3A* in myocytes due to phosphorylation of *FoxO3A* by activating *AKT*. *FoxO3A* overexpression or knockdown could reverse the specific effects of ACh on the expression of *VEGF-A/B*, *α/β-MHC*, *Glut4*, and *CPT1α*, sarcomere organization, glucose uptake and ATP production. Taken together, VNS optimized cardiomyocytes sarcomere organization and energy metabolism to improve heart function of the infarcted heart during the process of delaying and/or blocking the switch from compensated hypertrophy to decompensated heart failure, which were associated with activation of both P13K/AKT-FoxO3A-VEGF-A/B signaling cascade.

## Introduction

The chronic autonomic sympathetic/parasympathetic imbalance is a characteristic of cardiac disease, and reduced vagal activity is observed in heart failure (HF)^1^. Besides its anti-adrenergic and anti-arrhythmic activities^2^_,_ there has been growing attention on the vagus nerve and its neurotransmitter acetylcholine (Ach) because of Ach protective role in heart disease. These protective roles were associated with the anti-inflammatory and anti-apoptotic pathways, resulting in improved cardiac function and long-term survival in HF patients and HF animal models, that were a typical result of myocardium infarction (MI)^2–4^. However, whether or not VNS affects cardiomyocytes phenotype and energy metabolism during the heart repair remains to be determined.

Cardiomyocyte phenotype changes and energy metabolism indeed occur in a series of transitions from a healthy heart to compensated hypertrophy or even decompensated HF^5^. In the compensatory phase, hypertrophic responses, attributing to hypoxia inducible factor-1 (HIF-1)/vascular endothelial growth factors (VEGF-A), increase oxygen demand and promote myocardial angiogenesis to dissipate the hypoxic condition and to maintain cardiac contractile function^6^. In the pathological hypertrophied heart, sustained accumulation of p53 inhibited the transcriptional activity of HIF-1 that led to attenuation of angiogenesis and coronary flow reserve^7,8^, triggering progression of maladaptive HF^9^. Of interest, the unique effects of p53 are mainly evident in the HF model induced by adriamycin treatment^10^, mutations in the cardiac α-actin gene (Actc1)^11^, and pressure overload^9^, rather than in the myocardial infarction (MI) model by coronary artery ligation^12^. This indicates that an undefined mechanism coordinates myocardial growth and angiogenesis in the pathophysiology of cardiac hypertrophy and HF.

Cumulative evidence has shown that the lack of cardiomyocyte-secreted ACh leads to dysregulation in cardiac activity and dysfunction in cardiomyocyte remodeling^13,14^. Subsequent studies further demonstrated that the over-expression of cardiomyocytes’ vesicular ACh transporter (VACHT) or choline acetyltransferase (ChAT) increased ACh synthesis which protected the heart against ischemia and inhibited ventricular remodeling induced by sympathetic hyperactivity or angiotensin II (Ang II)^15–17^. Our recent study has shown that vagus nerve stimulation (VNS) improved angiogenesis, coronary blood flow (CBF), and heart function in the infarcted heart through activating VEGF-A/B^18^, and these specific effects could be markedly abolished by Flt1 blocker AMG706, similar to inhibition of angiogenesis in adaptive cardiac hypertrophy model by a decoy VEGF receptor, Flk1-Fc. More importantly, studies have shown that VEGFs were required to maintain cardiac function, which are associated with coronary microvessels, ventricular contractile function, and energy metabolism, especially both VEGF-A for glycolysis and VEGF-B for lipid metabilism^19–22^. These promising results indicate that the VNS-treated heart could need a new metabolic pattern to adapt to the beneficial coordination of angiogenesis and cardiac hypertrophy against the progression of HF.

In the present study, VNS increased α-MHC expressions, sarcomere organization, and ATP production through activating AKT/FoxO3A/VEGF signaling besides decreasing the levels of p53. We thus hypothesized that VNS promoted the improvement of cardiomyocytes phenotype and energy metabolism in the injured heart via the formation of an integrated signal system of ACh-m/nAChR-FoxO3A-VEGF-A/B.

## Method

### Animals, MI model and VNS

According to the Guide for the Care and Use of Laboratory Animals published by the US National Institutes of Health and China, animal studies were performed accordingly. Experimental Animal Centre of Hubei Medical University provided Sprague-Dawley (SD) rats (male, 250–300 g) that meet the criteria. The Institutional Animal Care and Use Committee of Hubei Medical University approved animal protocols.

According to the published protocol^18^, MI model was prepared by ligating the left anterior descending coronary artery (LAD) of rats. Briefly, after anaesthetized with ketamine (50 mg/kg, i.p.) and xylazine (10 mg/kg, i.p.), tracheal ventilation for rats with room air was carried out by using a Colombus ventilator (HX-300, Taimeng Instruments, Chengdu, China). Then the LAD was ligated after left lateral thoracotomy was performed at the fourth intercostal space. Lastly, MI occurrence was identified by observation of the injury demarcation with blanching of the myocardium as well as electrocardiography before chest closure.

Seven days after the ligation of LAD, survivors were randomized into groups of sham or active stimulation. In the actively stimulated group (VNS), the vagal nerve was stimulated with regular pulses of 0.2 ms duration at 20 Hz for 10 s every minute for 4 h^18^. In the sham group (MI), similar procedures were conducted without initiating the vagal nerve stimulation. The electrical voltage of pulses was optimized in each rat to obtain a 10% reduction in heart rate. To prevent drying and to provide insulation, the electrodes and the vagus nerve were immersed in a mixture of white petrolatum (vaseline) and paraffin.

To determine the role of mACh-R and α7-nAChR in VEGF expressions and cardiomyocytes phenotypes of the infarcted heart following the stimulation of VNS, mecamylamine (MLA, 10 mg/kg, i.p.) or atropine (Atrop, 10 mg/kg, i.p.) were performed 1 h (h) before VNS (six rats/group), as described previously^18^.

### Mitochondrial structure, function, actin assembly and sarcomere organization

Transmission electron microscopy was used to evaluate the role of VNS in mitochondrial function and sarcomere in the infarcted hearts 28 days after VNS treatment. Subsequently, MitoTracker^®^ Deep Red FM (M22426, Invitrogen, USA) and a lipophilic cationic probe JC-1 (M34152, Invitrogen, USA) were used to evaluate mitochondrial transmembrane potential (MMP) and mitochondrial mass^23^. ATP content were detected by ELISA. Furthermore, indirect immunofluorescence analysis was performed using anti-sarcomeric actin (α-Actinin, ab9465, Abcam) antibody as primary anti-body, and TRITC-conjugated anti-mouse IgG (Jackson ImmunoResearch) was used as secondary antibody^24^. For F-actin staining, red fluorescent phalloidin conjugate (ab112127, Abcam) was used. Images were taken with a microscope (Nikon.80i.JP). A total of 30 images/group within six repeats were taken by using the same imaging parameters. Images were analyzed by two pathologists using Image J (Java) software (National Institutes of Health, USA) in a double-blind manner. To quantify sarcomere organization, percentage of the cells with organized sarcomeres per high-power field were calculated by the formula = α-actinin positive cells numbers/total cells numbers. To quantify cardiomyocyte hypertrophy, percentage of the cells with actin assembly per high-power field were calculated by the formula = F-actin positive cells numbers/total cells numbers^25^.

### Cardiomyocyte phenotype and energy metabolism

Gene chip analysis, Western blot, real-time-PCR, immunostaining, and glucose uptake assay (ab136955) were used to evaluate cardiomyocyte phenotype and energy metabolism in vivo and in vitro. The primers used are shown in Supplementary Table 1. GO analysis are shown in Supplementary Tables 2 and 3. Pathway analysis are shown in Supplementary Table 4.

### Myotube formation of H9C2 or C2C12 myoblasts

Differentiation of H9c2 myoblasts in myotubes was induced by changing the culture medium from proliferation to differentiation medium at cell confluence. And the cells were maintained for at least 1 week in the differentiation medium contained DMEM supplemented with 2 mM l-glutamine, 100 IU/mL penicillin, 100 μg/mL streptomycin, 1% insulin–transferrin sodium selenite (Sigma, USA), and 1% FBS. The myoblast C2C12 cells were inoculated in 75 cm^2^ culture dish and cultured with high glucose DMEM containing 10% fetal bovine serum (FBS) at 37 °C and 5% CO_2_. When cells confluence reached 70–80%, the culture medium was replaced with high glucose DMEM containing 2% horse serum (HS) to induce C2C12 cell differentiation. Immunofluorescence staining of MyHC (sc-20641, 1:150, Santa Cruze), sarcomeric actin (α-Actinin, ab9465, Abcam) were used to evaluate traits of myotube^26^.

### Statistical analyses

Data shown are mean ± SD. Statistical significance between two groups was determined by paired or unpaired Student’s *t*-test. Results for more than two experimental groups were evaluated by one-way ANOVA to specify differences between groups. *P* < 0.05 was considered statistically significant.

Other detailed methods section are available in the Supplementary material online.

## Results

### VNS improved cardiac sarcomere structure and heart function in infracted heart

To efficiently prepare the therapeutic model of VNS in infarcted hearts, firstly, the left ventricle vagal innervation were confirmed by immunostaining of VACHT, the vagal nerves were disorganized decreased in the infracted hearts (Supplementary Fig. S1A and B). Along with the VNS, there was an increase in ACh levels in heart tissues and serum (Supplementary Fig. S1C), indicating that VNS caused cardiac vagus nerve to release more ACh. In addition to restoring autonomic balance, the anti-inflammatory effects of vagal activity may aid to halt or reverse the progression of HF, where inflammation is known to play a deleterious role^1^. As shown in Supplementary Fig. S2A–F, VNS successfully alleviated the typical inflammatory course triggered in infracted hearts. Finally, VNS decreased cell apoptosis, accompanied by increased pAKT levels, decreased p53 and p16 levels (Supplementary Fig. S2G–L), and a decline in the nuclear translocation of p65-NF-κB (Supplementary Fig. S2D) in infarcted hearts. These observations proved the successful establishment of the therapeutic model of VNS in infarcted hearts.

**Fig. 1:**
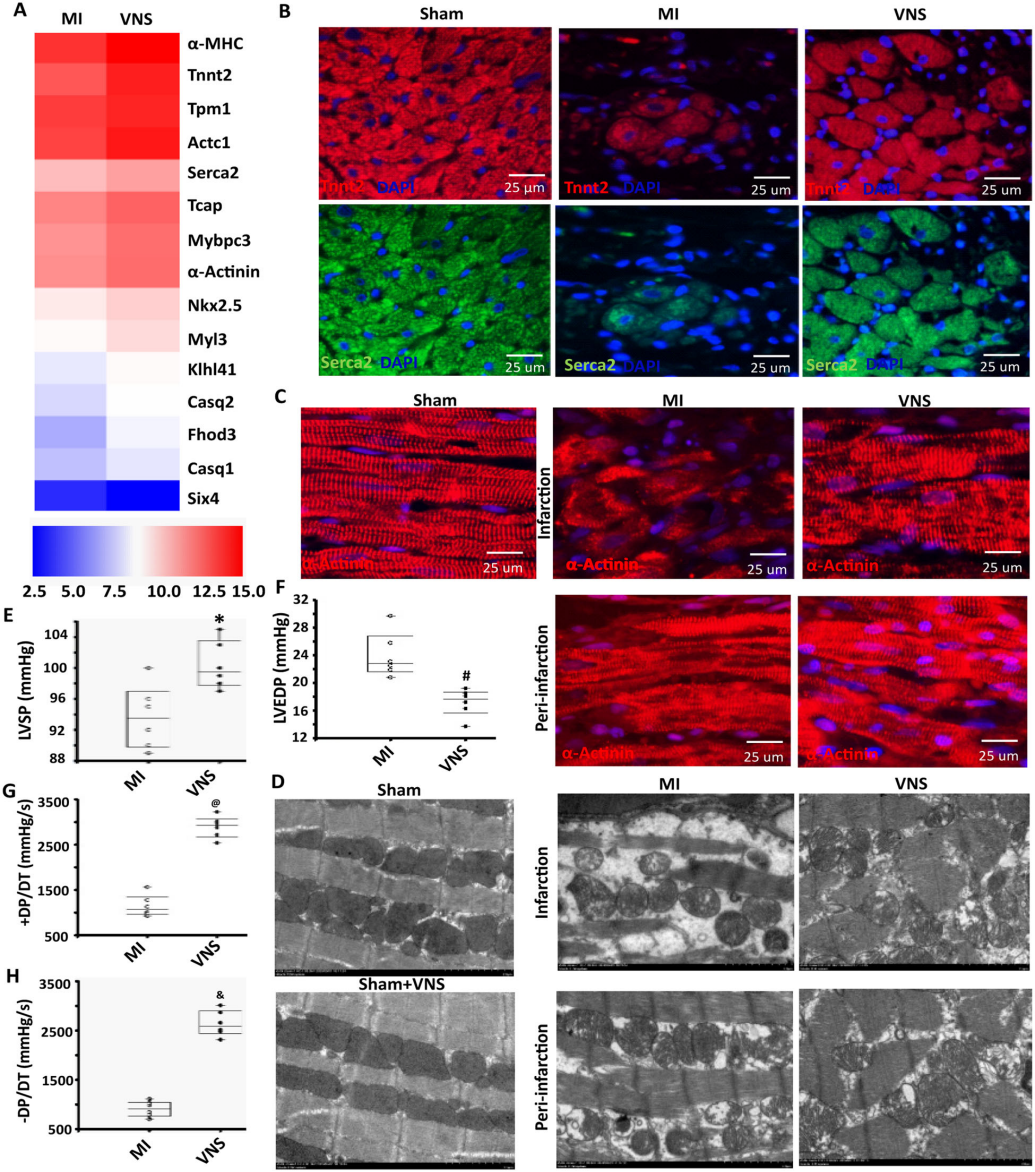
VNS improved cardiac sarcomere structure and heart function in infracted heart. **A** Gene chip thermography showed the obvious changes in genes-related cardiac sarcomere structure. **B** VNS increased the expressions of Tnnt2 and Serca2 in the infarcted heart as evaluated by IF. **C** VNS increased sarcomere organization in infraction and peri-infraction area of the infarcted heart as evaluated by sarcomeric alpha actinin (α-actinin) staining. **D** Typical image of transmission electron microscop. Scale: 2 μm. **E**–**H** VNS improved function of infarcted heart. *n* = 6, *P = 0.0110 vs. MI; ^#^*P* = 0.0023 vs. MI; ^@^*P* = 0.0000 vs. MI; ^&^*P* = 0.0000 vs. MI. For all scatter plots, data are mean ± SEM; unpaired Student’s *t*-test **E**–**H**.

To observe the role of VNS in the cardiac sarcomere structure and heart function in infarcted hearts, we performed gene expression microarray, the result of which is shown in Fig. 1A. Compared with mock-controls, VNS improved the cardiac sarcomere structure in infarcted hearts and increased conventional genes including *α-MHC*, *Serca2*, α-*A*c*tinin*, *Six4*, *Tnnt2*, *Nkx2-5*, *Tcap*, *Mybpc3*, *Tpm1*, *Casq1*, *Mylk3*, *Fhod3*, *Casq2*, and *Klhl41* (Fig. 1A and Supplementary Fig. S3A–C). Consistent with these changes, VNS also significantly increased the gene expressions of cardiac muscle contraction-related to cardiac sarcomere structure including α-*A*c*tinin*, Serca2, Tnnt2, and Tpm1, and improved sarcomere organization in infarcted hearts (Fig. 1B and C, and Supplementary Fig. S3A, B). Meanwhile, sarcomeres were broken, disorganized and distorted, mitochondria were swollen, vacuolated and fragmented in infarcted hearts under electron microscope, these specific changes could be effectively improved by VNS (Fig. 1D). Furthermore, LV function analysis showed that VNS increased LVSP, +d*P*/d*t*_max_, and –d*P*/d*t*_max_ of infarcted heart while decreasing the levels of LVEDP, indicating that VNS improved LV function compared to the MI animals (Fig. 1E–H). These results suggest that VNS improves cardiac sarcomere structure and heart function in the infarcted heart.

### VNS improved cardiomyocytes phenotype and sarcomere organization in the infarcted heart through activating m/n-AChR

**Fig. 2:**
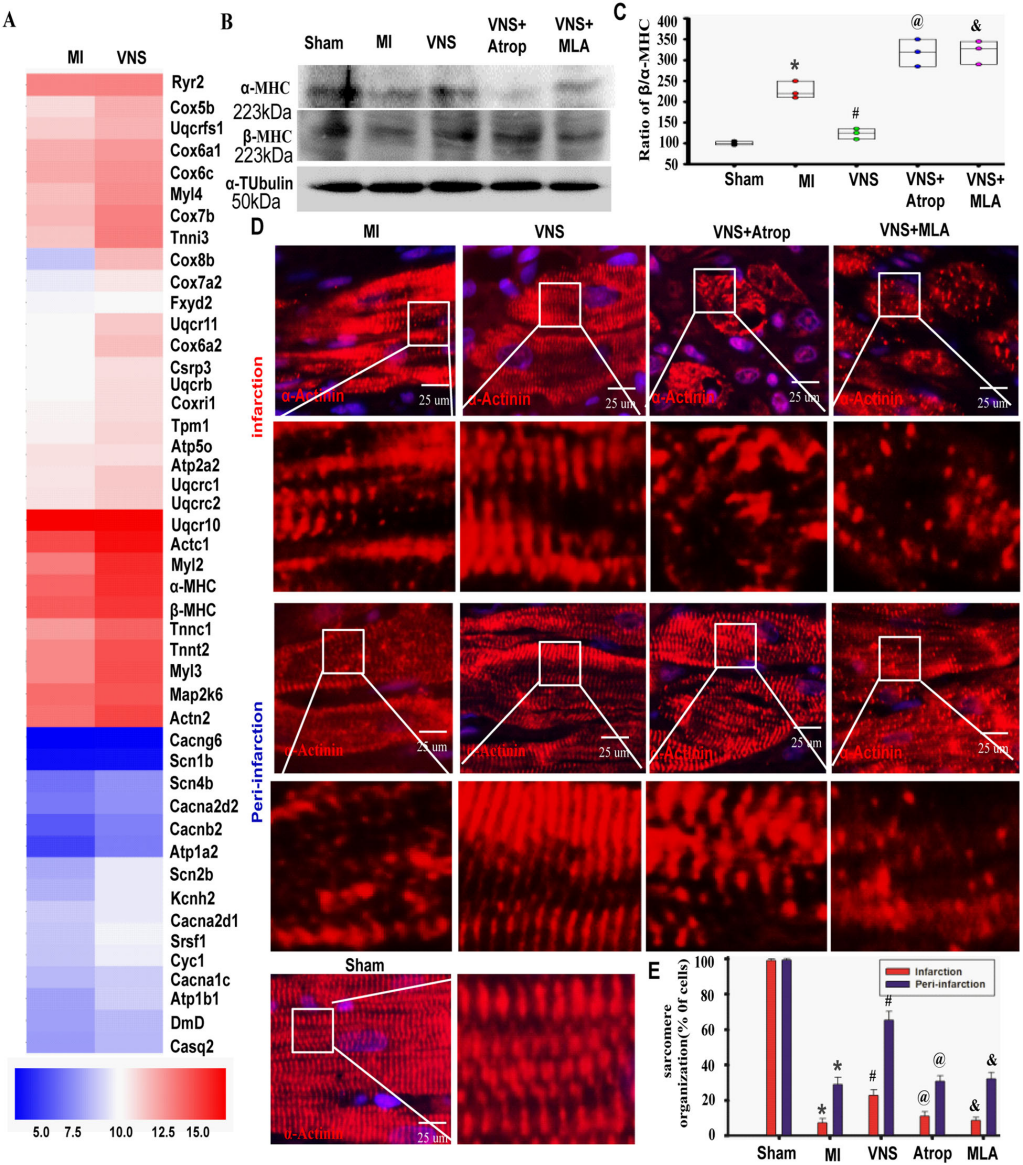
VNS improved cardiomyocytes phenotype and sarcomere organization in infracted heart through activating m/n-AChR. **A** Gene chip thermography showed the obvious changes in genes related “adult” heart in infarcted myocardium. **B** and **C** VNS increased the expressions of α-MHC in the infarcted heart as evaluated by western blot, and mAChR inhibitor atropine (Atrop) or α7-AChR blocker mecamylamine (MLA) markedly abolished the VNS-induced-α-MHC expression. *n* = 6, *P = 0.00684 vs. Sham; ^#^*P* = 0.00377 vs. MI. ^@^*P* = 0.00423vs. VNS; ^&^*P* = 0.00224 vs. VNS. **D** and **E** VNS increased sarcomere organization and optimized the sarcomere assembly in a disordered or punctate pattern of infraction and peri-infraction area of the infarcted heart as evaluated by α-Actinin staining, and mAChR inhibitor Atrop or α7-AChR blocker MLA markedly abolished the effect of VNS on cardiomyocyte sarcomere organization in infraction and peri-infraction area of the infarcted hearts. *n* = 6, *P < 0.05 vs. Sham; ^#^*P* < 0.05 vs. MI. ^@^*P* < 0.05 vs. VNS; ^&^*P* < 0.05 vs. VNS. For all scatter plots, data are mean ± SEM; one-way ANOVA with Bonferroni post hoc testing.

Because the cardiac sarcomere structure was associated with cardiomyocyte phenotype^27^, we used gene chip to analyze the traits of cardiomyocytes phenotype in MI heart treated with or without VNS. As shown in Fig. 2A, the expression of cardiomyocyte phenotype-associated genes, including *Uqcrb*, *Ryr2*, *Cacna1c, Cox7b*, *Cox5b*, *Cacng6*, *Myl2*, *Cyc1*, *Tpm1*, *Cacnb2*, *Fxyd2*, *Tnnt2*, *Myl3*, *Uqcr11*, *Cox6a1*, *Cox6a2*, *Uqcrfs1*, *Cox7a2*, *Uqcrc1*, *Atp1b1*, *Uqcr10*, *Cox8b*, *Atp2a2*, *Myl4*, *Uqcrc2*, *Actc1*, *Tnni3*, *Cox6c*, *Cacna2d2*, *α-MHC*, *Atp1a2*, *Cox4i1*, *Cacna2d1*, *Tnnc1*, *and β-MHC*, were analyzed (Fig. 2A, Supplementary Fig. S4B). The results showed that VNS drastically increased ‘adult heart’ α-MHC expressions and related sarcomere organization in infarcted heart, and the mACh-R and α7-nAChR blocker (Fig. 2B–E, Supplementary Figs. S5 and S6), respectively, could markedly abolish these specific effects. This indicates that VNS improved cardiomyocytes phenotype and sarcomere organization in the infarcted heart through activating m/n-AChR.

### VNS improved myocardium mitochondrial function and energy production

**Fig. 3:**
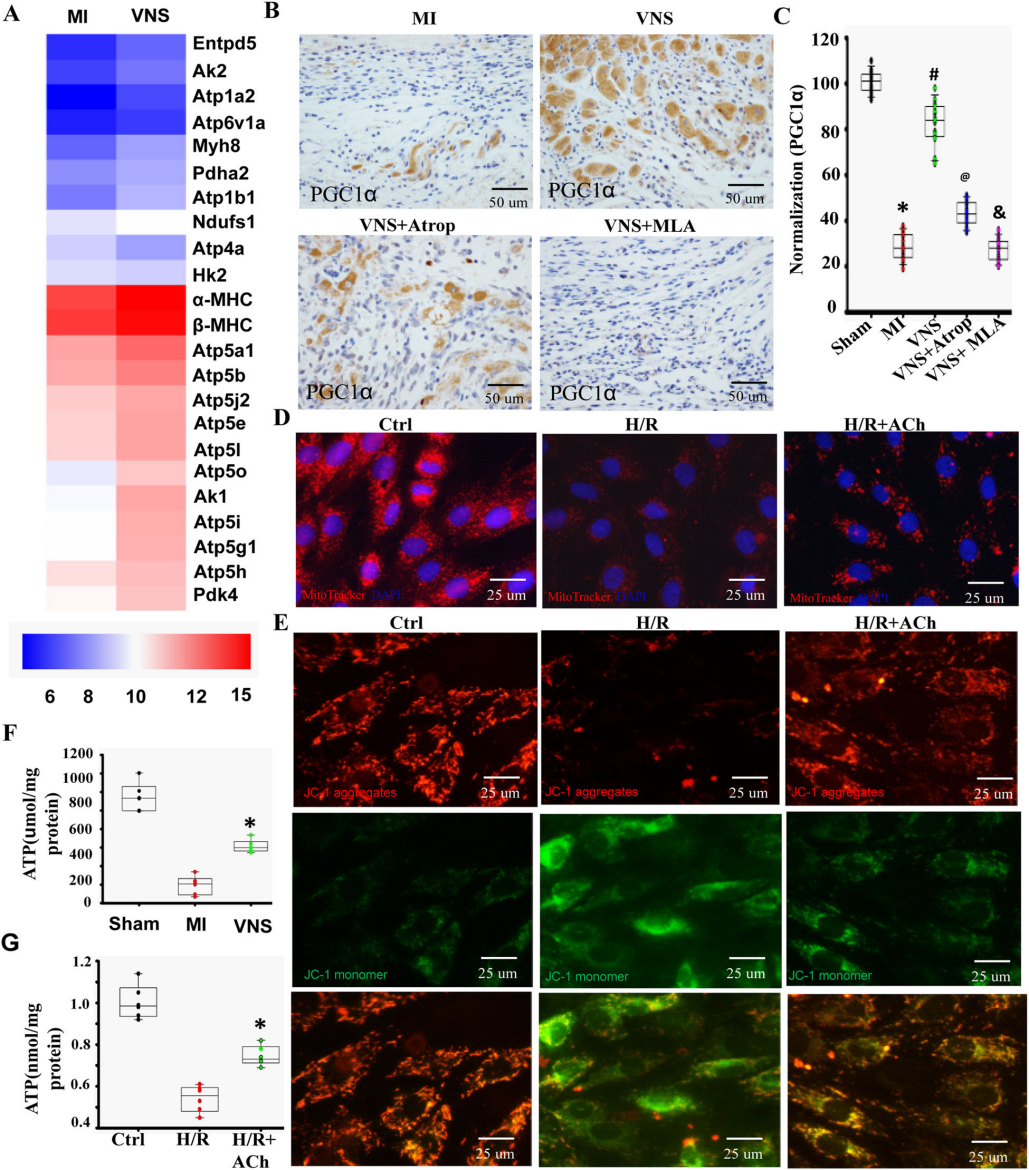
VNS improved myocardium mitochondrial function and energy production. **A** Using gene chip technology, cellular component including mitochondrion, mitochondrial inner membrane, mitochondrial respiratory chain complex I, and mitochondrial proton-transporting ATP synthase complex were markedly improved. Gene chip thermography showed the obvious changes in genes-related mitochondrial function. **B** and **C** VNS increased PGC1α expression in infraction area of the infarcted heart as evaluated by PGC1α staining, and mAChR inhibitor Atrop or α7-AChR blocker MLA markedly abolished the effect of VNS on PGC1α expression in infraction area of the infarcted hearts. *n* = 6, *P < 0.05 vs. Sham; ^#^*P* < 0.05 vs. MI. ^@^*P* < 0.05 vs. VNS; ^&^*P* < 0.05 vs. VNS. **D** ACh improved mitochondrial mass as evaluated by mean fluorescence intensity (MFI) using MitoTracker^®^ Deep Red FM staining. **E** ACh improved mitochondrial function as evaluated by ratio of red to green using JC-1staining. **F** VNS increased the levels of ATP in infarcted heart. *n* = 6, *P = 0.00001 vs. MI. **G** ACh increased the levels of ATP in H9C2 cells. *n* = 6, *P = 0.00012 vs. H/R. For all scatter plots, data are mean ± SEM; one-way ANOVA with Bonferroni post hoc testing.

Mitochondria, also known as the powerhouse of the cell, generate most of the ATP needed to fuel the body’s cells, including cardiomyocytes^27^. Gene chip analysis showed that VNS significantly improved ATP metabolic process (Supplementary Fig. S7), and some of the key genes including *Atp5j2*, *Entpd5*, *Atp5i*, *Ak2*, *Atp5e*, *Atp5g1*, *Atp5l*, *Myh8*, *Atp1a2*, *Atp5o*, *Atp6v1a*, *Atp5b*, *Atp1b1*, *Ndufs1*, *Atp5h*, *Ak1*, *Atp5a1*, *β-MHC*, and *α-MHC* (Fig. 3A, Supplementary Fig. S7B). Indeed, mitochondrial mass and respiratory capacity are markedly reduced in HF, which is associated with decreased expression of peroxisome proliferator-activated receptor γ coactivator-1α (PGC1α). As shown in Fig. 3B, C and Supplementary Fig. S8A, B, PGC1α levels were significantly increased in the VNS-treated hearts compared with MI hearts, and these favorable effects could markedly be abolished by mACh-R and α7-nAChR inhibitors. To determine whether the beneficial effects of VNS on cardiomyocytes phenotype and sarcomere organization were associated with similar recovery of mitochondrial mass, we quantified mitochondrial content in H/R H9c2 myoblast cells treated with ACh by using MitoTracker staining. We also performed JC-1 staining to detect mitochondrial damage. As expected, mitochondrial damage, characterized by more monomeric JC-1, was increased (Fig. 3E and Supplementary Fig. S8D), while the mitochondrial mass was markedly reduced in H/R H9c2 myoblasts cells compared to normal cells (Fig. 3D and Supplementary Fig. S8C). ACh partially recovered mitochondrial mass (Fig. 3D and Supplementary Fig. S8C) and the formation of aggregates of membrane potential-dependent JC-1 (Fig. 3E and Supplementary Fig. S8D), as typical markers of healthy cells.

To confirm if the favorable effects of VNS and ACh on mitochondrial damage contributed to increasing ATP production, we determined the cardiac ATP levels. Coinciding with the alterations in ATP metabolic process, ATP contents were significantly increased in both the VNS-treated hearts (Fig. 3F) in vivo and H/R-H9c2 cells in vitro (Fig. 3G), suggesting that VNS could improve myocardium mitochondrial function and energy production.

### VNS improved cardiomyocytes’ metabolic process in the infarcted heart

Previous studies have shown that the metabolic pattern of HF was not similar to the normal adult heart that is mainly composed of fatty acid oxidative decomposition, but rather to that of fetal heart that is mainly dominated by glucose metabolism^28,29^. To observe the relationship between cardiomyocytes phenotype and metabolic mechanisms, we performed gene chip analysis, and the results showed that VNS substantially enhanced metabolic pathways including glycolysis/gluconeogenesis and fatty acid degradation, especially in oxidative phosphorylation and citrate cycle (TCA cycle) (Supplementary Fig. S4A).

Actually, during HF development, cardiac substrate’s main fuel preference shifts from fatty acids to glucose, and further, one of the key features of the later stages of HF is the disruption of myocardial glucose uptake and utilization. These particular changes are associated with down-regulation of fatty acid transporter carnitine palmitoyltransferase 1-α (CPT1-α) and glucose transporter type 4 (GLUT4), and up-regulation of pyruvate dehydrogenase kinase 4 (PDK4). Pyruvate dehydrogenase is required for carbohydrate intermediates to enter the Krebs cycle and PDK4 inhibits this enzyme complex^25^.

**Fig. 4:**
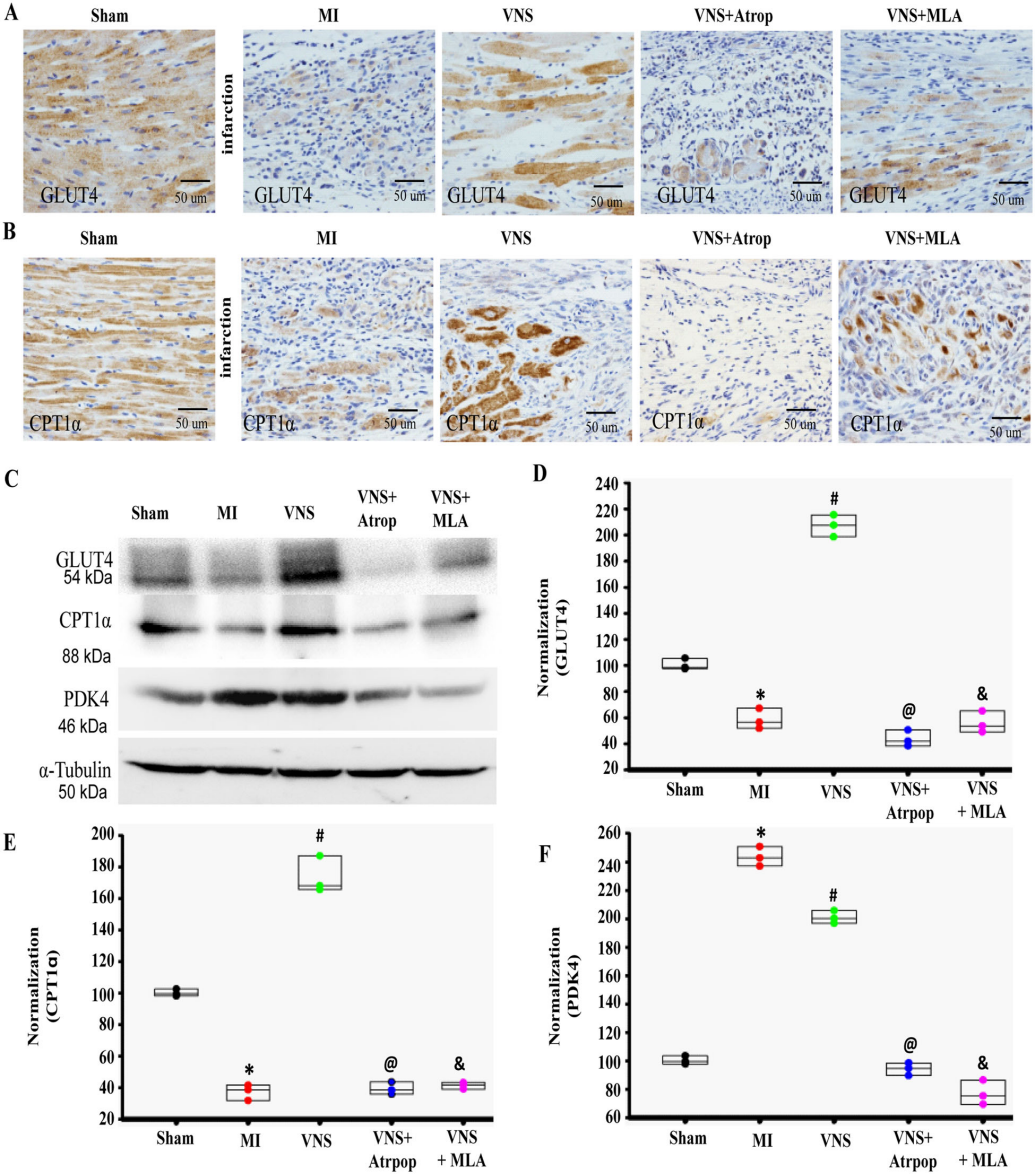
VNS improved cardiomyocytes metabolic process in infracted heart through m/n-AChR. **A** and **B** Typical image of CPT1α and GLUT4 in VNS-MI hearts with or without the treatment of mAChR inhibitor atropine (Atrop) or α7-AChR blocker mecamylamine (MLA) as determined by immunohistochemically staining. **C**–**F** VNS increased CPT1α and GLUT4 in infraction area of the infarcted heart as evaluated by western blot, and mAChR inhibitor Atrop or α7-AChR blocker MLA markedly abolished the effect of VNS on CPT1α and GLUT4 expressions in infraction area of the infarcted hearts. *n* = 3, *P < 0.05 vs. Sham; ^#^*P* < 0.05 vs. MI; ^@^*P* < 0.05 vs. VNS. ^&^*P* < 0.05 vs. VNS. For all scatter plots, data are mean ± SEM; one-way ANOVA with Bonferroni post hoc testing.

Correspondingly, our results show a decrease in CPT1-α and GLUT4 levels and an increase in PDK4 expression in cardiac tissue of the MI-heart compared to the sham group (Fig. 4). Interestingly, VNS treatment restored the expression of all these markers to the levels observed in the sham models (Fig. 4A–F, Supplementary Fig. S9), suggesting that VNS restores myocardial substrate metabolism. However, these effects could abolish the mACh-R and α7-nAChR blockers. It is thus plausible that these receptors are involved in the complex metabolic process.

### VEGF participates in VNS-mediated improvement of cardiomyocytes phenotype and sarcomere organization in the infarcted heart

**Fig. 5:**
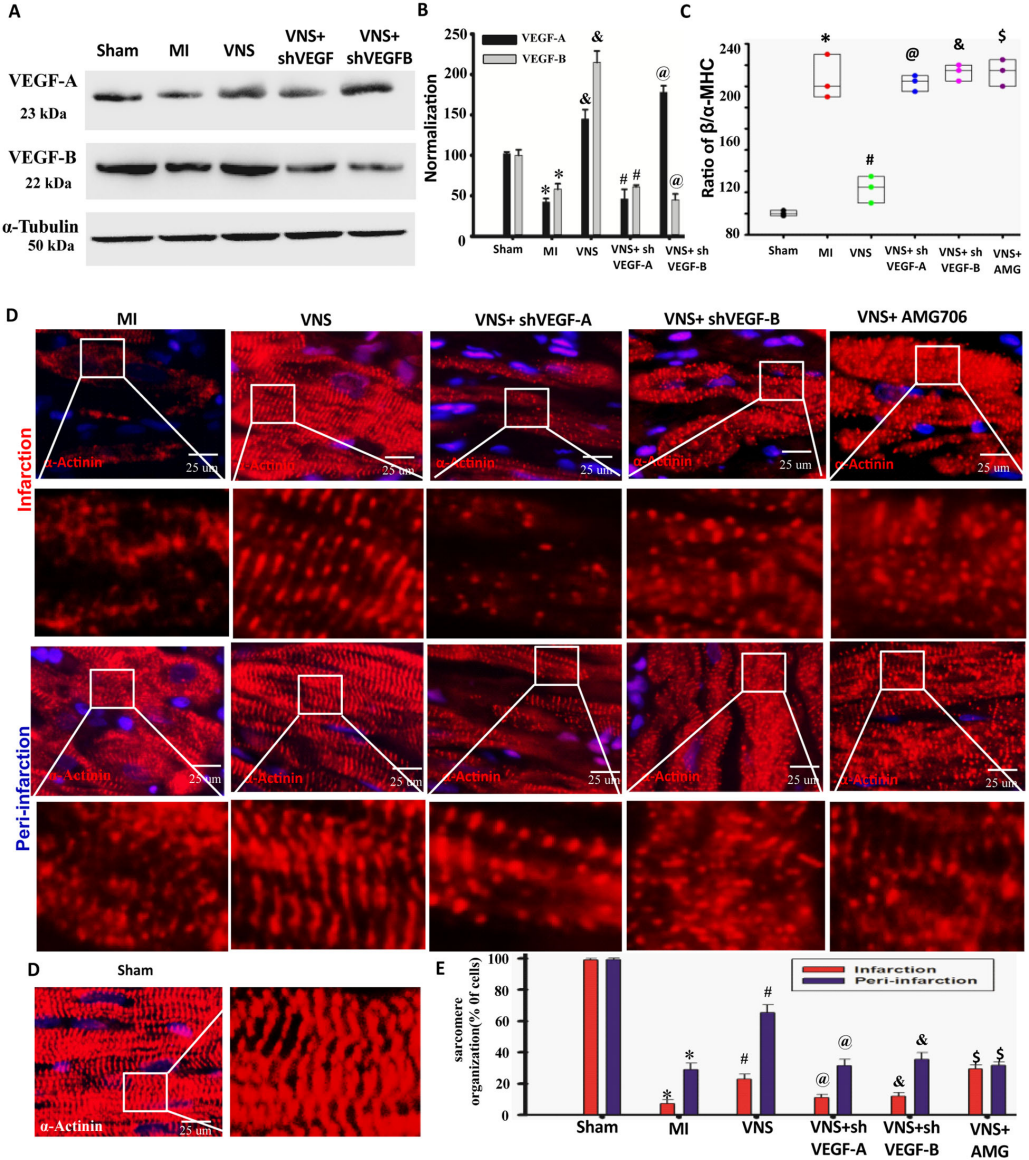
VEGF involved in VNS-mediated improvement of cardiomyocytes phenotype and sarcomere organization in infracted heart. **A**–**C** VNS promoted VEGF-A/B expression in the infarcted heart as detected by western blot **A** and semi-quantitative analysis **B**. *n* = 3,*P < 0.05 vs. Sham;^&^*P* < 0.05 vs. MI; ^@^*P* < 0.05 vs. VNS; ^#^*P* < 0.05 vs. VNS. **C** VNS-induced expression of α-MHC and β-MHC in infarction and peri-infarcted area of the infarcted heart, and the effective role could be obviously abrogated by VEGF-A/B shRNA or VEGFR1 blocker AMG706 as determined by α-MHC and β-MHC staining. *n* = 3,*P < 0.05 vs. Sham; ^#^*P* < 0.05 vs. MI; ^@^*P* < 0.05 vs. MI; ^&^*P* < 0.05 vs. VNS; ^$^*P* < 0.05 vs. VNS. **D** and **E** VNS increased sarcomere organization and optimized sarcomere assemble in infraction and peri-infraction area of the infarcted heart as evaluated by α-actinin staining, and the specific role could be obviously abrogated by VEGF-A/B shRNA or VEGFR1 blocker AMG706, showing more obviously disordered arrangement and point aggregation. *n* = 6,*P < 0.05 vs. Sham; ^#^*P* < 0.05 vs. MI; ^@^*P* < 0.05 vs. VNS; ^&^*P* < 0.05 vs. VNS; ^$^*P* < 0.05 vs. VNS. For all scatter plots, data are mean ± SEM; one-way ANOVA with Bonferroni post hoc testing.

VEGF is known to play a crucial role in coordinating myocardial growth and angiogenesis in the pathophysiology of cardiac hypertrophy and HF^6^. Moreover, our previous study has shown that VNS repairs the infarcted heart through inducing angiogenesis and arteriogenesis via VEGF-A/B^18^. To further explore whether VEGF participates in the improvement of cardiomyocytes phenotype and sarcomere organization by VNS, we firstly found that VNS promoted VEGF-A/B expressions in infarcted hearts (Fig. 5A, B). Secondly, VEGF-A or VEGF-B shRNA in vivo could eradicate the improved cardiomyocytes phenotype (Supplementary Figs. S10 and S11) and sarcomere organization (Fig. 5C–E) effects seen in infarcted hearts by VNS. In vitro, ACh markedly abolished the inhibitory effects of NE on differentiation and myotube formation of H9c2 myoblasts cells. More importantly, ACh-mediated effects on H9c2 myoblasts cells could be markedly reversed by the knockdown of VEGF-A or VEGF-B by shRNA (Supplementary Fig. S12). Altogether, these results suggest that VEGF-A/B participates in VNS-induced cardiomyocyte differentiation and sarcomere organization.

Consistent with the in vivo and in vitro findings, functional analysis in our previous study has shown that LV function, including LVSP, LVEDP, +d*P*/d*t*_max,_ and –d*P*/d*t*_max_ was significantly improved in VNS-treated hearts compared to the MI animals. Furthermore, VEGF shRNA reduced VNS-improved LV function^18^. These results demonstrate that VNS could improve heart function, attributing to the optimized cardiomyocytes phenotype and sarcomere organization in infarcted heart through VEGF signaling.

### VNS improved cardiomyocytes metabolic process in the infarcted heart through VEGF signaling

**Fig. 6:**
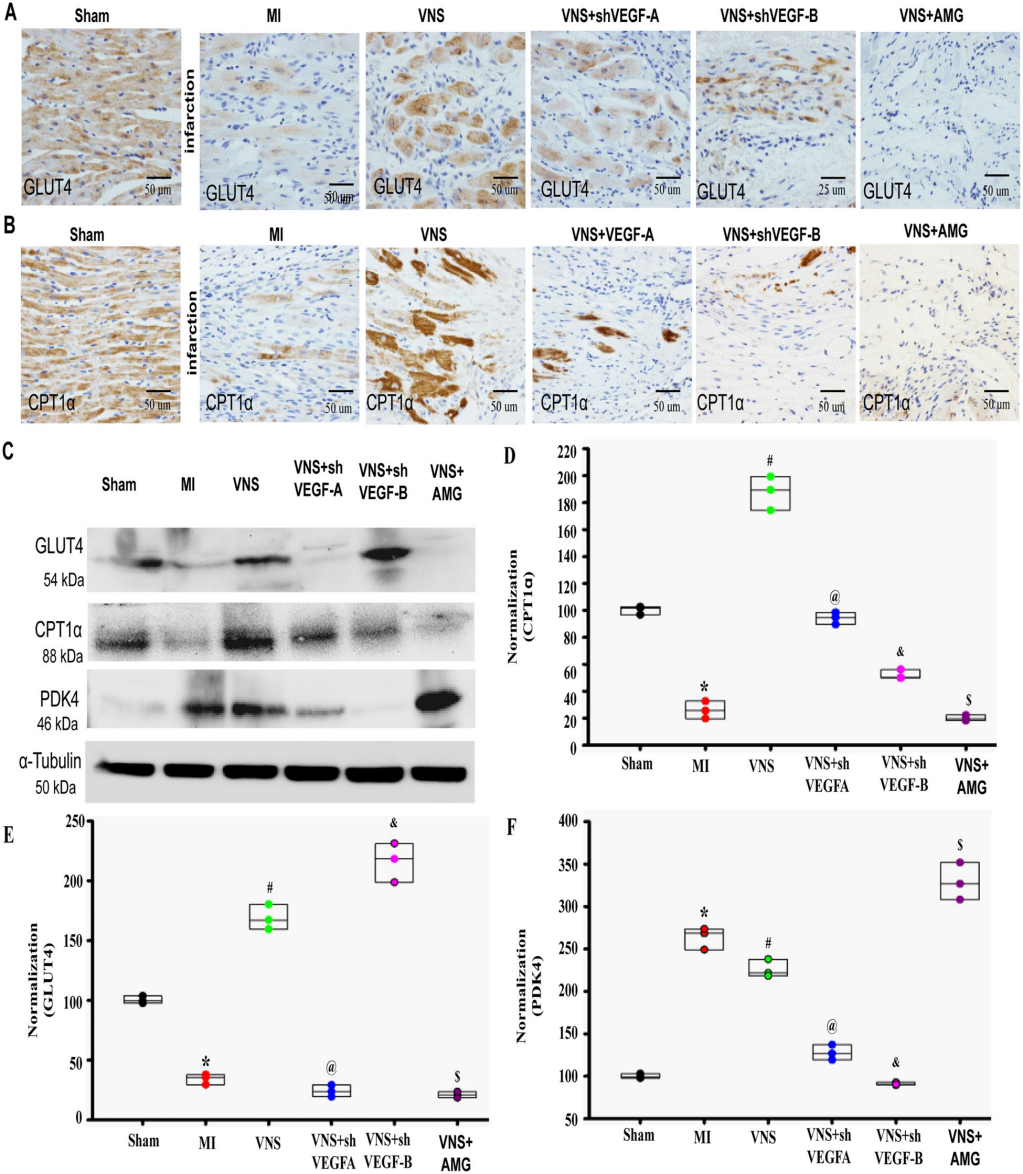
VNS improved cardiomyocytes metabolic process in infracted heart through VEGF signaling. **A** and **B** Typical image of CPT1α and GLUT4 in VNS-MI hearts with or without the treatment of knockdown of VEGF-A or VEGF-B by shRNA, or VEGFR1 blocker AMG as determined by immunohistochemically staining. **C**–**F** VNS increased CPT1α and GLUT4 in infraction area of the infarcted heart as evaluated by western blot, and knockdown of VEGF-A or VEGF-B by shRNA, or VEGFR1 blocker AMG markedly abolished the effect of VNS on CPT1α and GLUT4 expressions in infraction area of the infarcted hearts. knockdowning VEGF-A more obviously reduced GlUT4 expression in the infarcted heart compared to that by knockdowning VEGF-B. By contrast, knockdowning VEGF-B more obviously reduced CPT1ɑ expression in the infarcted heart compared to that by knockdowning VEGF-A and VEGFR1 blocker AMG reversed VNS-mediated both CPT1α and GLUT4 changes compared with that by knockdowning VEGF-A or VEGF-B. AMG effectively abolished the inhibition role of VNS in reducing PDK4 expression. However, knockdown of VEGF-A or VEGF-B by shRNA not only did not reverse, but also enhanced the inhibitory effects of VNS on PDK4. *n* = 3, ^*^P < 0.05 vs. MI; ^#^*P* < 0.05 vs. MI; ^@^*P* < 0.05 vs. VNS; ^&^*P* < 0.05 vs. VNS; ^$^*P* < 0.05 vs. VNS. For all scatter plots, data are mean ± SEM; one-way ANOVA with Bonferroni post hoc testing.

Because previous studies showed that VEGF-A and VEGF-B involved in myocardium energy metabolism^19–22^, we explored the involvement of ACh-induced VEGF in cardiomyocytes and its energy metabolism. As shown in Fig. 4, CPT1-α and GLUT4-levels were reduced, and PDK4 expression was increased in cardiac tissue of the MI-heart compared to the sham group. Interestingly, VNS treatment restored the expression of all these markers to the levels of those seen in the sham group, and these specific effects could be abolished by VEGF-R1 inhibitor AMG706, and the knockdown of VEGF-A or VEGF-B (Fig. 6A–F, Supplementary Fig. S13A–E). Moreover, VEGF-A knockdown in VNS-treated hearts resulted in a more obvious reduction of GLUT4, suggesting that VNS improved myocardial glucose metabolism through VEGF-A. Unlike VEGF-A, knockdown of VEGF-B significantly decreased CPT1-α in VNS-treated hearts, suggesting that VNS improved myocardial fatty acid metabolism through VEGF-B. Furthermore, in vitro, ACh-mediated effects on H9c2 myoblasts differentiation and myotube formation, and lipid deposition under H/R condition could be prominently reversed by the knockdown of VEGF-A or VEGF-B, especially VEGF-B knockdown, suggesting that ACh improved myocardial fatty acid metabolism through VEGF-B (Supplementary Fig. S13F, G). These results demonstrate that VNS-induced VEGF-A/B that is involved in enhancing cardiomyocyte metabolic process including glucose and fatty acid metabolism, consistent with “fetal” to “adult” alteration of myocyte type and sarcomere organization in the infarcted heart.

### FoxO3A involved in alteration of VNS-mediated cardiomyocytes phenotype, sarcomere organization and metabolic process

**Fig. 7:**
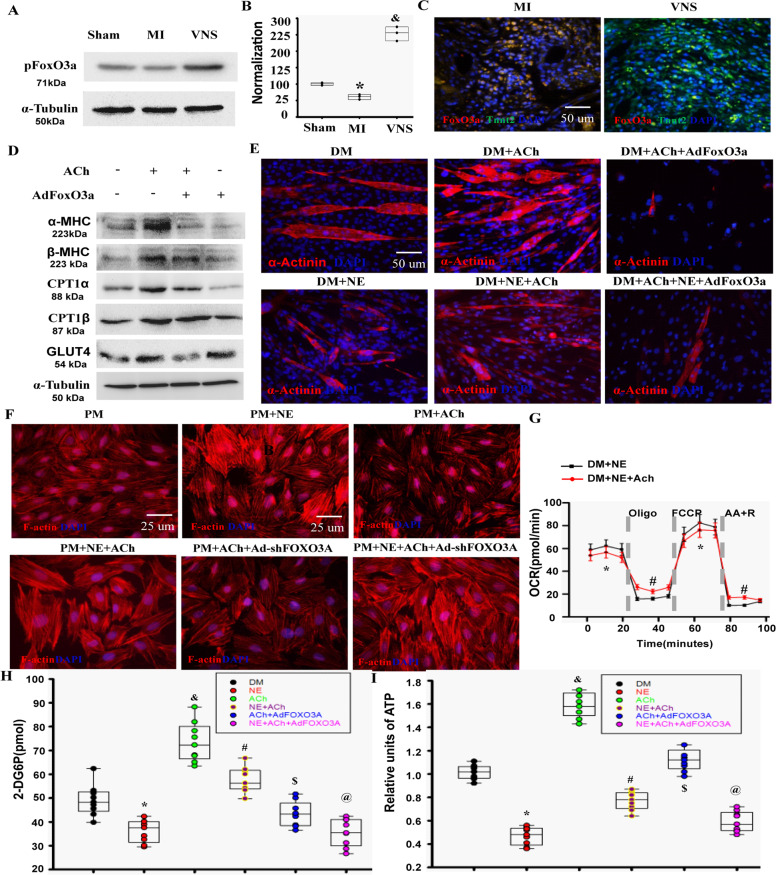
FoxO3A involved in alteration of VNS-mediated cardiomyocytes phenotype, sarcomere organization, and energy metabolism. **A** and **B** VNS increased the levels of pFoxO3A in the infarcted heart as evaluated by western blot **A**. **B** Semi-quantitative analysis of pFoxO3A as indicated. *n* = 3,*P < 0.05 vs. Sham; ^&^*P* < 0.05 vs. MI. **C** Typical image of decreased nuclear translocation by VNS in the infarcted heart. **D** ACh-induced expression of α-MHC, β-MHC, CPT1α, CPT1β, and GLUT4, the specific effects could be substantially abolished by over-expressing FoxO3A as determined by western blot. **E** ACh improved sarcomere organization in H9c2 myoblasts cells disturbed by NE under differentiation medium (DM), and the effective role could be obviously abrogated by over-expressing FoxO3A as determined by α-Actinin staining. **F** ACh reversed the enhanced effects of NE on sarcomere F-actin assembly in myocytes through FoxO3A. Typical image for F-Actin in H9c2 cells transfected with Ad-shFoxO3A (MOI:100) under proliferation medium (PM) with continuous 10^−5^ Mol/L NE and/or 10^−8^ Mol/L ACh as determined by F-Actin staining. **G** Oxygen consumption rates (OCR) at baseline and in the presence of oligomycin, FCCP [carbonyl cyanide *p*-(trifluoromethoxy) phenylhydrazone], and antimycin A + rotenone (AA + R). *n* = 3 independent experiments. *P < 0.05 vs. DM + NE. ^#^*P* < 0.05 vs. DM + NE. **H** ACh improved glucose uptake in H9c2 myoblasts cells inhibited by NE, and the effective role could be obviously canceled by over-expressing FoxO3A. **I** ACh increased ATP production in H9c2 myoblasts cells reduced by NE, which could be obviously reversed by over-expressing FoxO3A. *n* = 3, *P < 0.05 vs. DM; ^&^*P* < 0.05 vs. DM and NE; ^#^*P* < 0.05 vs. NE; ^$^*P* < 0.05 vs. ACh; ^@^*P* < 0.05 vs. NE + ACh. For all scatter plots, data are mean ± SEM; one-way ANOVA with Bonferroni post hoc testing.

It is known that FoxO3A represses VEGF expression in cancer cells^30^. To test whether this signaling pathway is involved in VNS-induced VEGF-A/B expression, we firstly detected the levels of FoxO3A and pFoxO3A in infarcted hearts. As shown in Fig. 7A–C, VNS increased the levels of pFoxO3A, leading to decreased nuclear translocation of FoxO3A in cardiomyocytes of infarction area in VNS-treated hearts (Fig. 7B). Similarly, ACh reduced the levels of nuclear translocation of FoxO3A in NE-treated H9c2 myoblasts cells (Supplementary Fig. S14). Indeed, FoxO3A, as a typical downstream target of PI3K/AKT, could be phosphorylated by pAKT^31^. Considering the results of PI3K-AKT signaling pathway (Supplementary Figs. S2, S4) and decreased FoxO3A levels (Fig. 7C) in VNS-treated hearts. We used PI3K-AKT pathway-specific inhibitors to block the signaling pathway mentioned above and determine whether ACh-induced VEGF expression can be altered. We found that the typical PI3K/AKT inhibitor wortmannin decreased the ACh-mediated FoxO3A phosphorylation as well as the VEGF-A and VEGF-B expressions (Supplementary Figs. S15, S16), suggesting PI3K-AKT signaling regulated their expression and release. Furthermore, the forced expression of FoxO3A markedly abolished the expression and release of VEGF mediated by AKT (Supplementary Fig. S16D–F). More importantly, mAChR inhibitor atropine and nAChR inhibitor mecamylamine, could reduce the levels of pAKT and pFoxO3a, abolishing the effects of expression and release of VEGF in H9c2 cells treated with ACh (Supplementary Fig. S16A–F). These data demonstrated that ACh, as a neurotransmitter of the vagus nerve, induced VEGF expression through m/n-AChR-PI3K/AKT-FoxO3A signaling pathway.

Coinciding with the role of VNS in altering cardiomyocytes phenotype and improving cardiomyocytes metabolic process in vivo, ACh induced higher expressions of α-MHC than that of β-MHC, improved sarcomere organization, and enhanced cardiomyocytes metabolic changes including Glut4, CPT1α, and CPT1β in vitro. Furthermore, PI3K/AKT inhibitor wortmannin (Supplementary Fig. S18) and the over-expression of FoxO3A (Fig. 7D, E, Supplementary Fig. S17) could significantly abrogate these particular effects.

Previous studies have shown that long-term overexcitement of sympathetic nerve released more NE, accompanied by decreased vagal activity during MI, could induce cardiomyocytes hypertrophy, leading to the loss of cardiomyocyte components^32^. By contrast, VNS could reduce sympathetic nerve activity and promote recovery of MI heart function^4^. As shown in Fig. 7E and Supplementary Fig. S19A, B, continuous delivery of NE inhibited differentiation, myotube formation and sarcomere organization of H9c2 myoblasts characterized by α-actinin, and Ach abolished these specific effects. Similarly, ACh abolished the inhibitory role of NE in differentiation and myotube formation of C2C12 myoblast cells and recovered the expression of MyHC1 and MyHC2x to normal levels (Supplementary Fig. S19C–F). More importantly, ACh-mediated effects on H9c2 myoblast cells could be markedly reversed by the overexpression of FoxO3A, suggesting that FoxO3A is involved in VNS-induced myocytes differentiation and sarcomere organization.

As a typical marker of cardiomyocytes hypertrophy, F-actin assembly was evaluated in H9c2 myoblast cells under proliferation medium. As shown in Fig. 7F and Supplementary Fig. S17F, continuous delivery of NE increased F-actin assembly in H9c2 myoblast cells, Ach could almost entirely abolish this specific role. Moreover, ACh-mediated effects on H9c2 myoblast cells could be enhanced by the knockdown of FoxO3A by shRNA, suggesting that FoxO3A participates in VNS-induced cardiomyocytes phenotype and sarcomere organization.

In the presence of 10^−8^ Mol/L ACh, NE-treated C2C12 myoblast cells under differentiation medium exhibited an increase in oxidative aerobic metabolism compared with that of NE + ACh treatment as demonstrated by a decrease in oxygen consumption (Fig. 7G).

To explore the role of ACh in myocytes metabolism, we assessed glucose uptake and ATP levels. As shown in Fig. 7H, I, we observed increased glucose uptake in ACh-treated H9c2 myoblast cells, and obviously reversed the inhibitory effects of NE in glucose uptake of the cells. Consistent with the changes in glucose uptake, ATP levels showed the similar changes following the treatment with ACh and/or NE in H9c2 myoblast cells. Furthermore, ACh-mediated effects on H9c2 myoblast cells could be markedly reversed by the overexpression of FoxO3A, suggesting that FoxO3A participates in VNS-induced myocytes metabolism optimization.

Altogether, these results suggested that VNS could recover the cardiomyocyte components related to myocardial contraction, possibly attributing to the FoxO3A signaling.

## Discussion

Our studies made three novel observations. Firstly, we find that VNS-induced VEGF-A/B expression cooperatively regulated cardiomyocytes phenotype, sarcomere organization, and metabolic process in the infarcted heart. Secondly, VNS-triggered cardiomyocytes phenotype switch improve energy metabolism related to left ventricular performance. Lastly, the VEGF-A/B expression is regulated by the ACh/m/nAChR/AKT/FoxO3A signal cascade.

VNS has emerged as a promising physiotherapy that reduces hospitalization of myocardial ischemic/infarction and HF patients, besides just autoimmune diseases^33^. Several exciting pre-clinical studies have demonstrated that VNS in the setting of HF results in diminished heart rate, improved heart function, reduced hypertrophy, preservation of intrinsic cardiac neuronal function, and increased survival^2–4,34,35^. Of interest, clinical trials of VNS in HF are controversial. Dicarlo’ study has shown improvement in left ventricular ejection fraction and end-systolic diameter with either left or right-sided VNS^36^. Gold and his colleagues, however, provided a puzzling report that VNS treatment for HF had no benefit in primary outcome, but showed improvements in the quality of life and 6-min walking distance^37^. These differences may be associated with stimulation methods, patient selection, and assessment way, in addition to disease models^38^.

**Fig. 8:**
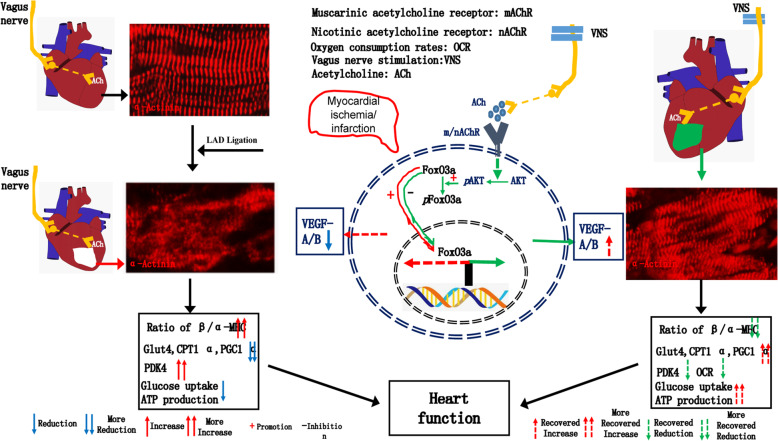
Working model. VNS optimized the levels of *α/β-MHC* and *α-Actinin* positivesarcomere organization in cardiomyocyte of infarcted heart.VNS improved glucose uptake, mitochondrial function, oxygen consumption and ATP production, matching the increase of Glut4 and CPT1α and the decrease of PDK4 in infarcted hearts. VNS optimized cardiomyocyte sarcomere organization and energy metabolism through activation of P13K/AKT-FoxO3A-VEGF-A/B signaling cascade.

As a crucial molecule for myocardial angiogenesis, VEGF expression was usually regulated by Hif-1 under hypertrophic stimuli or hypoxia/ischemia, and the series of events were terminated by p53 accumulation, causing the disruption of coordinated cardiac hypertrophy and angiogenesis, driving the transition to HF in pressure overload model except for LAD-induced MI model^6–11^. Similarly, myocardial angiogenesis and arteriogenesis, and β/α-MHC ratio were drastically improved in the MI-hearts following the treatment of VNS^18^. Meanwhile, the decreased p53 levels and increased VEGF-A/B expressions in VNS-treated MI-hearts were observed, accompanied by attenuated cardiac hypertrophy, in accord with the results that overexpression of VEGF-A or VEGF-B in myocardium did not promote cardiac hypertrophy^39^, or only induced physiological hypertrophy^21^, while contributing to neovascularization. Considering the existing evidence that genetic deletion of p53 did not effectively block the progression to HF^12^, microarray assay was analyzed, showing that PI3K/Akt signaling pathway were substantially altered in VNS-treated MI hearts. In line with the changes that short-term Akt1 activation-induced physiological hypertrophy via coordinated upregulation of VEGF expression by Hif-1α^6^, VNS and ACh triggered VEGF-A/B expressions, angiogenesis, and cardiomyocytes phenotype switch through activating Akt1, possibly resulting from the direct activation of ACh-induced Hif-1α by Akt1^17^, and/or the reduced p53 levels^9^. Herein, we should provide a novel mechanism that VNS-induced VEGF-A/B expression through inactivation of FoxO3A, as a result of the activation of upstream signaling PI3K/AKT. Also, combining the effect of VNS and ACh on reducing the p53 levels, another possible cause for the inactivation of FoxO3A by p53 could not be excluded, due to previous evidence that p53 transactivated FoxO3A^40^ and MDM2 acted downstream of p53 as an E3 Ligase to promote FOXO ubiquitination and degradation^41^. Therefore, VNS and ACh regulated VEGF-A/B expressions via the forming of an integrated signal system of p53–Akt–FoxO3A Fig. 8.

Cardiomyocytes, the most critical cellular unit of the myocardium, expressed several sarcomeric proteins, including myosin and actin; abnormal changes in the mature distribution pattern of actin assembly and sarcomere organization were significant causes of the heart pump’s dysfunction and failure^42^, which involved many molecules such as *Tpm1, Fhod3, MYBPC3*, *and TCAP*^16,43–48^. These genes expressions were increased in VNS-treated MI hearts, indicating that VNS was beneficial for the improvement of cardiac function and increase in supportive conditions that optimized both actin assembly and sarcomere organization in the infarcted heart. Furthermore, more accumulation of F-actin in cardiomyocytes under pathological conditions such as overloading, genotoxicity, or chemical toxicity could not only damage sarcomere organization but also hinder ATP production and utilization during the process of cardiomyocytes contraction and relaxation^45,49–51^. By contrast, VNS or ACh reduced F-actin accumulation in infarcted heart and injured cardiomyocytes by NE. The beneficial effects could be abolished by overexpressing-FoxO3A, indicating that VNS could improve heart function through an integral actin assembly and sarcomere organization coupling with energy metabolism.

Because of evidence that VNS improved sarcomere organization and heart function, we were propelled to explore whether VNS affects cardiac performance in the cardiomyocyte energy metabolism, due to an essential role of ATP in high-energy demanding organs, including the heart and liver. Indeed, the beneficial effects of VNS on glucose metabolism have consistently been observed in models of diabetes^52^. To some extent, this is the first report about the effects of VNS on cardiac energy metabolism. PGC1-α is a critical mediator of mitochondrial biogenesis and the reductions in PGC1-ɑ are thought to contribute to mitochondrial dysfunction observed in HF. Several studies have shown that VNS could improve the size and number of mitochondria in myocardial ischemia and diabetic hearts^53–56^. This is consistent with our observation that VNS restored mitochondria mass and membrane potential while recovering PGC1-ɑ expression, suggesting that the effects of VNS on mitochondrial function can be translated to non-progressive HF.

Furthermore, the changes in the expression levels of CPT1, GLUT4, and PDK4 observed suggest that the myocardial capacity to oxidize glucose and fatty acids is improved by VNS, especially under the condition of slightly reduced oxygen consumption, indicating that VNS restored myocardial glucose and fatty acid oxidation close to non-failling levels, leading to the increase of ATP levels. These results suggested that VNS-improved cardiomyocytes phenotype as a concomitant optimization in metabolic process. However, the relative contributions of glycolysis versus oxidative phosphorylation to ATP generation following the treatment of VNS in infarcted heart against the progress of HF should be clarified in further study.

Similar to previous studies that reported that VEGF-A/B could readjust endothelial cells and adipocytes metabolic pathways to favor uptake, transport and oxidation of fatty acids and glucose across and into cells^22,57,58^, besides inducing stem cell differentiation into cardiomyocytes^59–62^. Our results show that VNS and ACh-induced VEGF-A primarily involved in glucose uptake while induced VEGF-B mainly participated in lipid uptake in myocytes, suggesting that inactivated FoxO3A by VNS triggered the alteration of cardiomyocytes phenotype switch and corresponding metabolic improvement targeting VEGF-A/B.

Taken together, as for the prevention or treatment of human HF, VNS provides a novel and promising clinical strategy through optimized energy metabolism and sarcomere organization of myocardium during the process of delaying and/or blocking the transition from compensated hypertrophy to decompensated HF, which were associated with activation of both P13K/AKT–FoxO3A–VEGF-A/B signaling cascade.

## Supplementary information

Supplementary material

FIgure 1

Figure 2

Figure 3

Figure 4

Figure 5

Figure 6

Figure 7

Figure 8

Figure 9

Figure 10

Figure 11

Figure 12

Figure 13

Figure 14

Figure 15

Figure 16

Figure 17

Figure 18

Figure 19

Figure 20

Supplementary Table 1

Supplementary Table 2

Supplementary Table 3

Supplementary Table 4
