# Supplementary material for: Vagus nerve stimulation optimized cardiomyocyte phenotype, sarcomere organization and energy metabolism in infarcted heart through FoxO3A-VEGF signaling
Source: Cell Death Dis. 2020 Nov 12;11(11):971. doi: 10.1038/s41419-020-03142-0 (PMC7665220; doi:10.1038/s41419-020-03142-0)
Supplement: Supplementary file 1 — Supplementary material [file 41419_2020_3142_MOESM1_ESM.docx]

**ONLINE SUPPLEMENT**

**Vagus nerve stimulation optimized cardiomyocyte phenotype, sarcomere organization and energy metabolism in infarcted heart through FoxO3A-VEGF signaling**

Bin Luo^1,3#^, Yan Wu^1,3#^, Shu-lin Liu^1,2^, Xing-yuan Li^2^, Hong-rui Zhu^1^,Lei Zhang^2,3^,Fei Zheng^2^, Xiao-yao Liu^1^, Ling-yun Guo^2^, Lu Wang^2^, Hong-xian Song^2^, Yan-xia Lv^1,3^, Zhong-shan Cheng^4^, Shi-you Chen^5^, Jia-ning Wang^2,3^, Jun-ming Tang^1,2,3^*

^1^Department of Physiology, Hubei Key Laboratory of Embryonic Stem Cell Research, School of Basic Medicine Science, Hubei University of Medicine, Hubei, 442000, China.

^2^Institute of Clinical Medicine and Department of Cardiology, Renmin Hospital, Hubei University of Medicine, Shiyan, Hubei, 442000, China.

^3^Institute of Biomedicine, Hubei University of Medicine, Hubei, 442000, China.

^4^Applied bioinformatics center, St. Jude Children's Research Hospital, Memphis, Tennessee, United States

^5^The Department of Surgery, University of Missouri, Columbia, U.S.A.

^#^Co-first author

*Corresponding Author: Jun-ming Tang, Department of Physiology, Hubei Key Laboratory of Embryonic Stem Cell Research, Institute of Clinical Medicine, School of Basic Medicine Science, Hubei University of Medicine, Shiyan, Hubei 442000, China

Phone: 86-719-8875312

Email: tangjm416@163.com

**DETAILED METHODS**

**Animals**

According to the Guide for the Care and Use of Laboratory Animals published by the US National Institutes of Health and China, animal studies were performed accordingly. Experimental Animal Centre of Hubei Medical University provided Sprague-Dawley (SD) rats (male, 250–300 g) that meet the criteria. The Institutional Animal Care and Use Committee of Hubei Medical University approved animal protocols.

**Model establishment**

According to the published protocol^1^, myocardium infarction (MI) model was prepared by ligating the left anterior descending coronary artery (LAD) of rats. Briefly, after anesthetized with ketamine (50 mg/kg, i.p.) and xylazine (10 mg/kg, i.p.), tracheal ventilation for rats with room air was carried out by using a Colombus ventilator (HX-300, Taimeng Instruments, Chengdu, China). Then the LAD was ligated after left lateral thoracotomy was performed at the fourth intercostal space. At last, the occurrence of MI was identified by observation of the injury demarcation with blanching of the myocardium as well as electrocardiography before chest closure.

**Vagus nerve stimulation**

Seven days after the ligation of LAD, survivors were randomized into groups with sham or active stimulation. In the actively stimulated group (VNS), the vagal nerve was stimulated with regular pulses of 0.2ms duration at 20 Hz for 10 seconds every minute for 4 hours^1,2^. In the sham group (MI), similar procedures were conducted without initiating the vagal nerve stimulation. The electrical voltage of pulses was optimized in each rat to obtain a 10% reduction in heart rate. To prevent drying and to provide insulation, the electrodes and the vagus nerve were immersed in a mixture of white petrolatum (Vaseline) and paraffin.

To determine the role of mACh-R and α7-nAChR in VEGF expressions and cardiomyocytes phenotypes of the infracted heart following the stimulation of VNS, mecamylamine (MLA, 10 mg/kg, ip) or atropine (Atrop, 10 mg/kg, ip) were performed 1 hour (h) before VNS (six rats/group), as described previously^1^.

**Knockdown of VEGF-A/B *in vivo***

Construction of VEGF short hairpin RNA (shRNA) adenoviral vector were prepared as previously described^1^, the shRNA sequences were: VEGF-A165: GAGTTAAACGAACGTACTTGCAGA TGTGA; VEGF-B for rat: AGATGCACAA

ATCAGATGGTG; VEGF-B for human: AAUUUCCUGUCACGACACUUCGGU


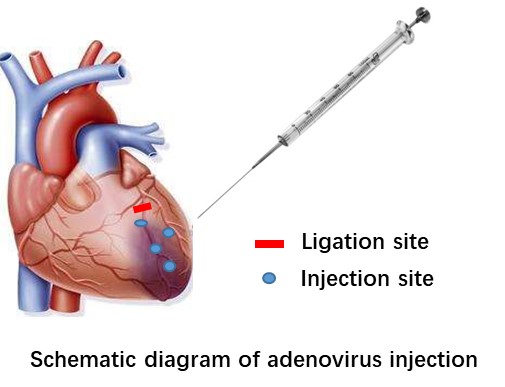
CUG. To confirm the role of VEGF-A or VEGF-B on cardiomyocytes in the infracted heart, local injection of Ad-shCtrl, Ad-shVEGF-A, or Ad-shVEGF-B (1×10^9^pfu in 200 μl) into the infarcted hearts (four sites, 50 μl per site, 12 rats/group) with a 30-gauge tuberculin syringe 3 day before the VNS. Two injections were in the myocardium bordering the ischemic area and two within the ischemic area^18^. Penicillin (150,000 U/mL, i.v.) was given before each procedure. Buprenorphine hydrochloride (0.05 mg/kg, s.c.) was administered twice a day for the first 48 hours after the procedure.

**Measurement of hemodynamic parameters**

Hemodynamic parameters were measured 28 days after each treatment as described previously^18^. In brief, hemodynamic parameters including left ventricular systolic pressure (LVSP), left ventricular end-diastolic pressure (LVEDP), and rate of rise and fall of ventricular pressure (+dP/dt_max_ and –dP/dt_max_) were measured simultaneously using BL-420s (Chengdu Tai-meng, Co, China). The heart was rapidly removed for other analyses after the measurements.

**H9c2 cell culture, hypoxia-reoxygenation and groups**

To further confirm if the role of ACh in VEGF was involved in cardiomyocytes, H9c2 cells were cultured. m/n-AChR expression in H9c2 cells were performed by immunofluorescence staining.

To further addressed the possible signaling mechanism of the ACh-induced VEGF-A/B expressions in cardiomyocytes, **mACh-R inhibitor a**tropine (1μM), nACh-R inhibitor mecamylamine (MLA, 10^−4^ M), or PI3K/AKT inhibitors wortmannin (50 nM) were pretreated for 1 hour, then treated with ACh (10^-5^ M) for 24 hours. These cells were harvested and lysed in RAPI buffer with protease and phosphatase inhibitors for western blot.

H9c2 cells were cultured in a low-oxygen condition (95% N2 + 5% CO_2_) for 21 h in a humidified hypoxia chamber. After hypoxia incubation, the medium was replaced with or without the addition of ACh, and the cells were exposed to normal-oxygen condition (95% air + 5% CO_2_) for reoxygenation for 6 h^2,3^. Control cells were cultured in normoxic conditions. The cells were collected separately for further analysis.

**Measurement of mitochondrial mass and membrane potential**

Mitochondrial mass was analyzed using a far red-fluorescent dye that stains mitochondria in live cells, MitoTracker® Deep Red FM (M22426, Invitrogen, USA)^4^. Mitochondrial transmembrane potential (MMP) was evaluated using a sensitive fluorescent dye, a lipophilic cationic probe JC-1 (M34152, Invitrogen, USA) as previously described^5^. H9c2 cells were grown on cover slips followed by H/R with ACh treatment, and then incubated with 5 mM JC-1 dye or 1 mM MitoTracker at 37°C for 15 min. The cells were washed with PBS and analyzed immediately with a fluorescent microscope.

**Myotube formation of H9C2 myoblasts**

Differentiation of H9c2 myoblasts in myotubes was induced by changing the culture medium from proliferation to differentiation medium at cell confluence. And the cells were maintained for at least 1 week in the differentiation medium contained DMEM supplemented with 2 mM L-glutamine, 100 IU/mL penicillin, 100 μg/mL streptomycin, 1% insulin-transferrin sodium selenite (Sigma, USA) and 1% FBS^4,5^.

**Metabolic assays**

Oxygen consumption rate) was measured using the XFe96 analyzer (Seahorse Bioscience). For NE and ACh experiments, 5x10^4^ C2C12 myoblasts cells were added per well in 6 XFe96 microplates. C2C12 myoblasts cells were cultured under differentiation medium (2% HS+high glucose-DMEM) with continuous 10^-5^ Mol/L NE and/or 10^-8^ Mol/L ACh for 6 days. The continuous exposure was to add the same dose of NE or ACh each day when the differentiation medium was replaced. Myotube formation was analyzed 6 days after the NE and/or ACh treatment.

Oxygen consumption rates were measured sequentially under basal conditions, in response to 3 uM oligomycin (an ATP synthase inhibitor), 1 uM FCCP (a mitochondrial uncoupler), and 4 μg/mL of antimycin A with 3 μM rotenone (both potent inhibitors of oxidative phosphorylation)^6^.

The ATP Assay Kit from Abcam (#ab83355, Abcam, CN) was used to detect the ATP levels of heart tissues and cells according to the manufacturer’s instructions. Results were then normalized by protein concentrations of each test.

**Actin Assembly and Sarcomere organization**

Indirect immunofluorescence analysis was performed using anti-sarcomeric actin (α-Actinin, ab9465, Abcam.) antibody as primary anti-body, and TRITC-conjugated anti-mouse IgG (Jackson ImmunoResearch) was used as secondary anti-body^2^. For F-actin staining, red fluorescent phalloidin conjugate (ab112127, Abcam.) was used. Images were taken with a microscope (Nikon.80i.JP.). A total of 30 images/group within six repeats were taken by using the same imaging parameters. Images were analyzed by two pathologists using Image J (Java) software (National Institutes of Health, USA) in a double-blind manner. To quantify sarcomere organization, percentage of the cells with organized sarcomeres per high-power field were calculated by the formula=α-Actinin positive cells numbers/total cells numbers. To quantify cardiomyocyte hypertrophy**,** percentage of the cells with actin assembly per high-power field were calculated by the formula=F-actin positive cells numbers/total cells numbers^7^

**Glucose Uptake Assay**

Glucose uptakes in the differentiated H9c2 myoblasts were evaluated according to protocol of Glucose Uptake Assay Kit (ab136955).

**Western blot**

Western blot was carried out with primary antibody against VEGF-B (1:1000, Abcam, Abcam, ab51867), VEGF-A (1:1000, Abcam, ab46154), AKT(1:1000, Cell Signaling, #9272s), pAKT (1:1000, Cell Signaling, #9271s), α-MHC (1:500, Abcam, 224046), β-MHC (1:500, Santa Cruz, sc-168678), α-Actinin (1:500, Abcam, ab9465), pFoxO3A(1:500, Santa Cruz, sc-101683), FoxO3A(1:500, Santa Cruz, sc-11351), CPT1α (1:1000, Abcam, ab83862), CPT1β (1:1000, Abcam, ab134988), GLUT4 (1:1000, Abcam, ab654), PDK4(1:1000, Abcam, ab89295), p53(1:1000, Abcam, ab26), p16 (1:1000, Abcam, ab51243), and α-tubulin (T9026, 1:5000, Sigma). Rat left ventricles were removed and grinded in liquid nitrogen. The samples were collected and homogenized on ice in a 0.1% Tween-20 homogenization buffer containing protease inhibitors. 50 µg of proteins were resolved in 10% SDS-PAGE gel and transferred onto a polyvinylidene fluoride (PVDF) membrane (Millipore). After being blocked with 5% nonfat milk, the membrane was incubated with primary antibody (1:1000 dilutions) for 90 min followed by incubation with horseradish peroxidase (HRP)-conjugated secondary antibodies (1:10000, Jackson ImmunoResearch). Protein expression was visualized by enhanced chemiluminescence reaction and quantified by densitometry^8^.

**Immunostaining**

Heart tissues were immersion-fixed in 4% paraformaldehyde and embedded in paraffin. Serial transverse sections (5 μm) were cut across the entire long axis of the heart and mounted on slides. After dewaxing, hydration and heat-induced antigen retrieval, heart specimens were incubated in a blocking buffer (PBS containing 5% goat serum and 0.1%Triton X-100) at room temperature for 1 h. Incubations in antibodies (diluted 1:250 in blocking buffer) were carried out at 4°C overnight for primary antibodies, and room temperature for 2 h for secondary antibodies. The primary antibodies used were: rabbit anti-rat VAChT (No.139 103; 1:250; Synaptic Systems); NF-KBp65 (1:200, Santa Cruz, SC-109), rabbit-anti-rat VEGF-B (1:200, Abcam, ab185696), rabbit-anti-rat VEGF-A (1:200, Abcam, ab46154), Tnnt2 (1:500, Sigma, SAB210823), Serca2 (1:500, Abcam, ab2861), α-Actinin (1:500, Abcam, ab9465), Tropomyosin 1 (alpha)(1:1000, Abcam, ab47003ab133292), α-MHC (1:500, Abcam, 224046), β-MHC (1:500, Santa Cruz, sc-168678), MyHC (sc-20641, 1:150, Santa Cruze) and FoxO3A(1:500, Santa Cruz, sc-11351). The secondary antibodies were horseradish peroxidase (HRP)-labeled goat anti-mouse IgG, goat-anti-rabbit IgG, FITC-conjugated anti-rabbit IgG, or TRITC-conjugated anti-mouse IgG (Jackson ImmunoResearch), respectively^8^.

**Gene chip analysis**

Seven days after MI, the rats were treated with VNS. The hearts were collected for gene chip analysis (Oebiotech, Shanghai, China) 3 days days after the stimulation of VNS.

**Cardiomyocyte apoptosis assay *in vivo***

Cardiomyocyte apoptosis were evaluated as previously described^9^. In brief, three days after MI, the hearts were prepared for histopathological analysis. Serial transverse Sections (5 μm) were blocked with buffer [PBS containing 1 % fetal calf serum (FCS) and 0.1 % Triton X-100] at room temperature for 1 h, and then detected using the methods described in the manual of the In-Situ Apoptosis Detection Kit (MM_NF-S7165#, Millipore, USA). The number of apoptotic cells within random five fields each section was manually counted by two pathologists who were unaware of the experimental design. The ratio of cell apoptosis was calculated as the percentage of all cells per high power field (25 μm).

**ELISA**

ELISA for ACh was performed in cardiac tissue and serum treated with cholinesterase inhibitor eserine (100 uM) after VNS using a commercial kit by following the manufacture’s protocol (ab65345, Abcam). ELISA for TNFα (900-M73, PeproTech) and IL-1β (900-M91, PeproTech) were performed in cardiac tissue and serum after VNS using a commercial kit by following the manufacture’s protocol (ab65345, Abcam). ELISA for VEGF-A were carried out in H9c2 cells supernatant (Neobiosciece, China).

**Oil Red O staining**

Oil Red O was purchased from Sigma-Aldrich Corp. (St Louis, IL). Staining was performed as previously described^10^. After 7 days under differentiating conditions, media was removed from 6-well plates and rinsed once with PBS. Cells were fixed in 4% formaldehyde solution for 30 minutes at room temperature. Fixed cells were then washed with PBS and stained for 10 min using Oil red-O stain working solution at room temperature. After washing with distilled water five times and rinsing with PBS for 15 min, the whole well photographs were acquired with a camera. The numbers of Oil Red O in myotubes were calculated.

**The culture and differentiation of C2C12 cells**

The myoblast C2C12 cells was inoculated in 75cm^2^ culture dish and cultured with high glucose DMEM containing 10% fetal bovine serum (FBS) at 37 C and 5% CO_2_. When cells confluence reached 70% to 80%, the culture medium was replaced with high glucose DMEM containing 2% horse serum (HS) to induce C2C12 cell differentiation. The C2C12 cell differentiation into myotubes were observed every day under a microscope. The formation of myotubes from C2C12 cells were detected by myotube markers at the third, fifth and eighth day of differentiation ^11^.

**Immunofluorescence Staining**

Primary antibodies against MyHC (sc-20641, 1:150, Santa Cruze), M1-AChR (sc-7471, 1:150, Santa Cruze)，M2-AChR (sc-7472, 1:150, Santa Cruze)，M3-AChR (sc-9108, 1:150, Santa Cruze)，M4-AChR (sc-9109, 1:150, Santa Cruze)，M5-AChR (sc-9110, 1:150, Santa Cruze) and α7-nAChR (sc-5544, 1:150, Santa Cruze) were added into the corresponding wells, and incubated overnight at 4 °C. After washing with PBS 3 times for 15 min, cells were incubated with corresponding fluorescent dye-labeled secondary antibodies (1:250) at room temperature for 2 hours. DAPI (Molecular Probes) was used to stain nuclei. The corresponding images were observed and photographed under a fluorescence microscope ^12^.

**Myotube morphology**

Cells were stained for MyHC using MyHC polyclonal rabbit anti-mice antibody (sc-20641, 1:150, Santa Cruze) followed by anti-rabbit TRITC-labeled secondary antibody (Jackson Lab, 1:400, USA). DAPI was used to stain nuclei. Myotube was defined as 3+ nucleuses within a cellular structure in order to rule out myoblast cells undergoing mitosis. The images of five locations including up, down, left, right sides and center of each slide were photographed using an 80i Nikon fluorescent microscope (Nikon, Japan). A total of 30 images/group within six repeats were taken by using the same imaging parameters. Images were analyzed by two pathologists using Image J (Java) software (National Institutes of Health, USA) in a double-blind manner ^13^.

**Quantitative RT-PCR**

Total RNA was extracted from C2C12 cells or cardiac tissues using TRIzol reagents (Life Technologies) and transcribed into cDNA using the SuperScript II cDNA kit (Life Technologies). Quantitative PCR was performed using SYBR green PCR master mix (Applied Biosystems) in RotorGene 6000 Real-Time PCR System (Qiagen, Mannheim, Germany). The transcript levels of interested genes in the corresponding groups were compared after normalization to GAPDH levels ^14^. The primers used were shown in Supplemental.file.Table1 ^15^.

**Statistical analyses**

Data shown are mean ± SD. Statistical significance between two groups was determined by paired or unpaired Student’s *t*-test. Results for more than two experimental groups were evaluated by one-way ANOVA to specify differences between groups. *P<*0.05 was considered statistically significant.

**References**

1.Lv YX, Zhong S, Tang H, Luo B, Chen SJ, Chen L, Zheng F, Zhang L, Wang L, Li XY, Yan YW, Pan YM, Jiang M, Zhang YE, Wang L, Yang JY, Guo LY, Chen SY, Wang JN, Tang JM. VEGF-A and VEGF-B Coordinate the Arteriogenesis to Repair the Infarcted Heart with Vagus Nerve Stimulation. *Cell Physiol Biochem.* 2018; **48**:433-449.

2.Zhao M, He X, Bi XY, Yu XJ, Gil Wier W, Zang WJ. Vagal stimulation triggers peripheral vascular protection through the cholinergic anti-inflammatory pathway in a rat model of myocardial ischemia/reperfusion. *Basic Res Cardiol*. 2013; **108**:345.

3.Tang JM, Wang JN, Zhang L, Zheng F, Yang JY, Kong X, Guo LY, Chen L, Huang YZ, Wan Y, Chen SY. VEGF/SDF-1 promotes cardiac stem cell mobilization and myocardial repair in the infarcted heart. *Cardiovasc Res.* 2011; **91**:402-11.

4.Zhou R, Yazdi AS, Menu P, Tschopp J. A role for mitochondria in NLRP3 inflammasome activation. *Nature*. 2011; **469**:221-5.

5.van den Eijnde SM, van den Hoff MJ, Reutelingsperger CP, van Heerde WL, Henfling ME, Vermeij-Keers C, Schutte B, Borgers M, Ramaekers FC. Transient expression of phosphatidylserine at cell-cell contact areas is required for myotube formation. *J Cell Sci.* 2001; **114**:3631-42.

6.Zahalka AH, Arnal-Estapé A, Maryanovich M, Nakahara F, Cruz CD, Finley LWS, Frenette PS. Adrenergic nerves activate an angio-metabolic switch in prostate cancer. *Science.* 2017; **358**:321-326.

7.Seguchi O, Takashima S, Yamazaki S, Asakura M, Asano Y, Shintani Y, Wakeno M, Minamino T, Kondo H, Furukawa H, Nakamaru K, Naito A, Takahashi T, Ohtsuka T, Kawakami K, Isomura T, Kitamura S, Tomoike H, Mochizuki N, Kitakaze M. A cardiac myosin light chain kinase regulates sarcomere assembly in the vertebrate heart. *J Clin Invest.* **117**:2812-24(2007).

8.Samniang B, Shinlapawittayatorn K, Chunchai T, Pongkan W, Kumfu S, Chattipakorn SC, KenKnight BH, Chattipakorn N.Vagus Nerve Stimulation Improves Cardiac Function by Preventing Mitochondrial Dysfunction in Obese-Insulin Resistant Rats. *Sci Rep*. 2016; **6**:19749.

9.Chen XG, Lv YX, Zhao D, Zhang L, Zheng F, Yang JY, Li XL, Wang L, Guo LY, Pan YM, Yan YW, Chen SY, Wang JN, Tang JM, Wan Y. Vascular endothelial growth factor-C protects heart from ischemia/reperfusion injury by inhibiting cardiomyocyte apoptosis.*Mol Cell Biochem.* 2016; **413**:9-23.

10.Tang JM, Yuan J, Li Q, Wang JN, Kong X, Zheng F, Zhang L, Chen L, Guo LY, Huang YH, Yang JY, Chen SY. Acetylcholine induces mesenchymal stem cell migration via Ca2+ /PKC/ERK1/2 signal pathway. *J Cell Biochem*. 2012; **113**:2704-13.

11.Chen ShJ, Xiang Li, Jiang M, et al. Isoprenaline induced muscle atrophy by inhibiting C2C12 cell differentiation into skeletal muscle cells. *Chin J Cell Biol*, 2017; **39**:1178-1187.

12.Ross JA, Levy Y, Svensson K, et al. SIRT1 regulates nuclear number and domain size in skeletal muscle fibers. *J Cell Physiol*. 2018; **233**:7157-716.

13.Millay DP, O'Rourke JR, Sutherland LB, et al. Myomaker is a membrane activator of myoblast fusion and muscle formation. *Nature*. 2013; **499**:301-5.

14.Chen X, Wan J, Yu B, et al.PIP5K1α promotes myogenic differentiation via AKT activation and calcium release. *Stem Cell Res Ther*. 2018; **9**:33.

15.Chen SJ, Yue J, Zhang JX, Jiang M, Hu TQ, Leng WD, Xiang L, Li XY, Zhang L, Zheng F, Yuan Y, Guo LY, Pan YM, Yan YW, Wang JN, Chen SY, Tang JM. Continuous exposure of isoprenaline inhibits myoblast differentiation and fusion through PKA/ERK1/2-FOXO1 signaling pathway. *Stem Cell Res Ther*. 2019; **10**:70.

**Supplemental Figures**

**sFIgure1. Vagus nerves changes in myocardium infarction model**

Myocardium infarction showed the deceased levels of VAChT + parasympathetic nerve with the trait of disarranged nerve fiber. (A) Immunostaining of VAChT for parasympathetic nerve. Green color: VAChT; Blue color: DAPI-labeled nuclei. (B) Semi-quantitative analysis for VAChT + parasympathetic nerve in hearts. n=6, **P*＜0.05 vs. Sham.

**sFigure.2. VNS suppressed inflammatory response and promoted cardiomyocytes survival in myocardium infarction model.**

VNS suppressed inflammatory response and promoted cardiomyocytes survival in myocardium infarction model. (A) Immunostaining of CD68 for macrophages. (B) Semi-quantitative analysis for CD68 + macrophages in hearts. n=6, **P*＜0.05 vs. Sham. (C) Gene chip assay for inflammatory response in infarcted hearts tissues treated with VNS, showing that VNS inhibited inflammatory response and NF-KB activity. (D)VNS decreased the levels of nuclear NF-KB p65 in MI hearts as evaluated by immunostaining of NF-KB p65. Red color: NF-KB p65; Blue color: DAPI-labeled nuclei. (E-F) VNS decreased the levels of TNF-α and IL-1β in serum of rats as detected by ELISA. (G-J) VNS reduced the levels of p53 and p16 while increasing pAKT levels in infarcted hearts as determined by western blot and semi-quantitative analysis. *P＜0.05 vs. MI, n=3. (K-L) VNS reduced numbers of cells in infarcted hearts as detected by TUNNEL and semi-quantitative analysis. *P＜0.05 vs. MI, n=6.

**sFigure.3. VNS improved cardiac muscle contraction-related cardiac sarcomere structure in infracted heart.**

Gene chip assay for cardiac muscle contraction-related to cardiac sarcomere structure, sarcomere organization in infarcted hearts treated with VNS, showing that VNS improved cardiac muscle contraction, sarcomere organization and assembly in infarcted hearts. (B)VNS increased the expressions of cardiac sarcomere structure and sarcomere organization in the infracted heart as evaluated by real-time PCR. *P＜0.05 vs. MI, n=3.

**sFigure4. VNS improved cardiomyocyte phenotype and metabolic process in infracted heart**

Gene chip assay for metabolic process in infarcted hearts treated with VNS, showing that VNS improved metabolic process in infarcted hearts. (B)VNS increased the expressions of cardiac metabolic process in the infracted heart as evaluated by real-time PCR. *P＜0.05 vs. MI, n=3.

**sFigure.5. VNS improved cardiomyocytes phenotype in infracted heart.**

(A) Immunostaining of ɑ-MHC in hearts. (B-C) Semi-quantitative analysis for ɑ-MHC in hearts. n=6, **P*＜0.05 vs. Sham; ^#^*P*＜0.05 vs. VNS; ^&^*P*＜0.05 vs. VNS. (D) Immunostaining of β-MHC in hearts. (E-F) Semi-quantitative analysis for β-MHC in hearts. n=6, **P*＜0.05 vs. Sham; ^#^*P*＜0.05 vs. VNS; ^&^*P*＜0.05 vs. VNS.

**sFigure.6. VNS improved cardiac muscle contraction-related cardiac sarcomere structure in infracted heart.**

(A-B) Immunostaining of Tpm1 in hearts. (C-D) Semi-quantitative analysis for Tpm1 in hearts. n=6, **P*＜0.05 vs. Sham; ^#^*P*＜0.05 vs. VNS; ^&^*P*＜0.05 vs. VNS.

**sFigure.7. VNS improved myocardium mitochondrial function and energy production.**

VNS improved myocardium mitochondrial function and energy production. (A) Using gene chip technology, cellular component including mitochondrion, mitochondrial inner membrane, mitochondrial respiratory chain complex I, and mitochondrial proton-transporting ATP synthase complex were markedly improved. (B)VNS increased the expressions of myocardium mitochondrial function and energy production in the infracted heart as evaluated by real-time PCR. *P＜0.05 vs. MI, n=3.

**sFigure.8. VNS improved myocardium mitochondrial function and energy production**

VNS improved myocardium mitochondrial function and energy production. (A-B) VNS increased PGC1α expression in peri-infraction area of the infarcted heart as evaluated by PGC1α staining, and mAChR inhibitor Atrop or α7-AChR blocker MLA markedly abolished the effect of VNS on PGC1α expression in peri-infraction area of the infarcted hearts. n=6, **P＜0.05* vs. MI; ^#^*P＜0.05* vs. VNS. ^&^*P＜0.05* vs. VNS. (C)ACh improved mitochondrial function by JC-1staining. n=6, **P＜0.05* vs. H/R.(D)ACh improved mitochondrial masss by MitoTracker® Deep Red FM staining. n=6, **P＜0.05* vs. H/R.

**sFigure.9. VNS improved cardiomyocytes metabolic process in infracted heart through m/n-AChR.**

VNS improved cardiomyocytes metabolic process in infracted heart through m/n-AChR. (A-B) VNS increased the expressions of CPT1ɑ and GLUT4 in the peri-infarcted heart as evaluated by immunohistochemical staining, and mAChR inhibitor atropine or α7-AChR blocker mecamylamine markedly abolished the effects of VNS on the indicated protein expressions. (C-D) Semi-quantitative analysis by detecting the gray value of CPT1ɑ and GLUT4 expressions in infarction area and peri-infarction area following the treatment of VNS with or without either atropine or MLA. n=6,*P＜0.05 vs. MI; ^&^P＜0.05 vs. VNS; ^#^P＜0.05 vs. VNS.

**sFigure.10. VEGF involved in VNS-mediated improvement of cardiac muscle contraction-related cardiac sarcomere structure in infracted heart**

(A)VNS promoted cardiac muscle contraction-related gene expressions in infarction and peri-infarcted area of the infarcted heart, and the effective role could be obviously abrogated by VEGF-A/B shRNA or VEGFR1 blocker AMG706 as determined by Tpm1 staining. (B-C)Semi-quantitative analysis by detecting the gray value of Tpm1 expressions in infarction area and peri-infarction area following the treatment of VNS with or without either shVEGFA, shVEGFB or AMG706. n=6,*P＜0.05 vs. MI; &P＜0.05 vs. VNS; #P＜0.05 vs. VNS; ^$^P＜0.05 vs. VNS.

**sFigure.11. VEGF involved in VNS-mediated improvement of cardiomyocytes phenotype in infracted heart through VEGF signaling.**

VEGF involved in VNS-mediated improvement of cardiomyocytes phenotype in infracted heart through VEGF signaling. (A) Immunostaining of ɑ-MHC in hearts. (B-C) Semi-quantitative analysis for ɑ-MHC in hearts. n=6, **P*＜0.05 vs. Sham; ^#^*P*＜0.05 vs. VNS; ^&^*P*＜0.05 vs. VNS; ^$^*P*＜0.05 vs. VNS. (D) Immunostaining of β-MHC in hearts. (E-F) Semi-quantitative analysis for β-MHC in hearts. n=6, **P*＜0.05 vs. Sham; ^#^*P*＜0.05 vs. VNS; ^&^*P*＜0.05 vs. VNS; ^$^*P*＜0.05 vs. VNS.

**sFigure.12. ACh involved in VNS-mediated improvement of cardiomyocytes phenotype in infracted heart.**

ACh involved in myotubes formation of H9c2 cells. (A)typical image of m1-5 and α7-nAChR expression in H9c2 cells. (B-C) ACh abolished the induced expressions of ɑ-MHC in myotubes of H9c2 under differentiation medium (DM).n=9,*P＜0.05 vs. DM; ^&^P＜0.05 vs. 10^-5^ Mol/L NE; ^#^P＜0.05 vs. 10^-8^ Mol/L ACh; ^$^P＜0.05 vs. 10^-8^ Mol/L ACh; ^@^P＜0.05 vs. 10^-5^ Mol/L NE+10^-8^ Mol/L ACh.

**sFigure.13. VNS improved cardiomyocytes metabolic process in infracted heart through VEGF signaling**

VNS improved cardiomyocytes metabolic process in infracted heart through VEGF signaling. (A-B) Typical image of CPT1α and GLUT4 in VNS-MI hearts with or without the treatment of knockdown of VEGF-A or VEGF-B by shRNA, or VEGFR1 blocker AMG as determined by immunohistochemical staining. (C-D) VNS increased the relative ratio of CPT1α and GLUT4 in peri-infraction area of the infarcted heart as evaluated by gray value of immunohistochemically staining, and knockdown of VEGF-A or VEGF-B by shRNA, or VEGFR1 blocker AMG markedly abolished the effect of VNS on CPT1α and GLUT4 expressions in peri-infraction area of the infarcted hearts. n=3, *P＜0.05 vs. MI; ^#^P＜0.05 vs. VNS;^&^P＜0.05 vs. NS;^$^P＜0.05 vs. VNS. (E)Typical image for Oil Red O staining of myotubes of H9c2 cells under differentiation medium following the continuous treatment of 10^-5^ Mol/L NE with or without 10^-8^ Mol/L ACh, accompanied by knockdown of VEGF-A or VEGF-B by shRNA. (F) ACh abolished the inhibitory effect of 10^-5^ Mol/L NE on myotube formation of H9c2 cells under differentiation, and the specific role could be obviously reversed by the knockdown of VEGF-A or VEGF-B. (G) ACh abolished the role of 10^-5^ Mol/L NE in increasing lipid levels within myotubes from H9c2 cells under differentiation medium, and the specific effects could be obviously reversed by the knockdown of VEGF-A or VEGF-B.

**sFigure.14. ACh decreased nuclear translocation of FOXO3a in NE-treated H9c2 cells**

(A-B) Nuclear translocation of FOXO3a in H9c2 cells under proliferation medium. ACh (10^-8^ Mol/L) reversed nuclear translocation of FOXO3a in NE (10^-5^ Mol/L) -treated H9c2 cells. n=9, *P＜0.05 vs.PM; ^&^P＜0.05 vs. NE; ^#^P＜0.05 vs. PM.

**sFigure.15. VNS induced VEGF expression in cardiomyocytes of infracted heart**

(A-C) VNS promoted VEGF-A/B expression in the infarcted heart as detected by immunohistochemically staining(A), western blot (B) and semi-quantitative analysis (C). n=3,*P＜0.05 vs. MI; &P＜0.05 vs. MI. (D) ACh dosage-dependently induced expression of VEGF-A and VEGF-B in H9c2 cells as determined by western Blot and semi-quantitative analysis (E-F). n=3,*P＜0.05 vs. Ctrl and 10^-5^ Mol/L ACh; ^&^P＜0.05 vs. 10^-5^ Mol/L ACh; ^#^P＜0.05 vs. 10^-4^ Mol/L ACh.

**sFigure16. FOXO3A involved in ACh-induced expression of VEGF in H9c2 cells.**

(A-C) ACh increased levels of pFoxO3a in H9c2 cells as detected by western blot (A) and semi-quantitative analysis (B-C). n=3,*P＜0.05 vs. Ctrl; ^&^P＜0.05 vs. 10^-5^ Mol/L ACh; ^#^P＜0.05 vs. 10^-5^ Mol/L ACh. (D) Overexpressing-FOXO3A in H9c2 cells abolished ACh-induced expression of VEGF as detected by western blot (D) and semi-quantitative analysis (E-F). n=3,*P＜0.05 vs. Ctrl; ^&^P＜0.05 vs. 10^-5^ Mol/L ACh; ^#^P＜0.05 vs. Ctrl. (G) mAChR inhibitor atropine or a7-nAChR inhibitor mecamylamine abolished ACh-induced expression of VEGF as detected by western blot. n=3,*P＜0.05 vs. Ctrl; ^&^P＜0.05 vs. 10^-5^ Mol/L ACh; ^#^P＜0.05 vs. Ctrl. (H) mAChR inhibitor atropine or a7-nAChR inhibitor mecamylamine, or overexpressing-FOXO3A in H9c2 cells abolished ACh-induced the release of VEGF as detected by Elisa. n=3,*P＜0.05 vs. Ctrl; ^&^P＜0.05 vs. 10^-5^ Mol/L ACh; ^#^P＜0.05 vs. 10^-5^ Mol/L ACh;^$^P＜0.05 vs. 10^-5^ Mol/L ACh.

**sFigure17. FOXO3A involved in alteration of ACh-mediated cardiomyocytes phenotype and sarcomere organization**

(A-D) ACh induced expression of α-MHC, β-MHC, CPT1α, CPT1β and GLUT4, the specific effects could be substantially abolished by over-expressing FOXO3A as determined by western blot and semi-quantitative analysis. n=3,*P＜0.05 vs. Ctrl; ^&^P＜0.05 vs. ACh; ^#^P＜0.05 vs. ACh. (E)ACh improved sarcomere organization disturbed by NE, and the effective role could be obviously abrogated by over-expressing FOXO3A as determined by α-Actinin staining. n=9, *P＜0.05 vs. DM; ^$^P＜0.05 vs. DM; ^&^P＜0.05 vs. NE; ^@^P＜0.05 vs. ACh; ^#^P＜0.05 vs. ACh+NE.(F) ACh reversed the enhanced effects of NE on F-actin assembly in H9c2 cells through FOXO3A. H9c2 cells transfected with Ad-shFOXO3A (MOI:100) under proliferation medium (PM) with continuous 10-5 Mol/L NE and /or 10^-8^ Mol/L ACh for 6 days were determined by F-Actin staining. n=9, *P＜0.05 vs. DM; ^@^P＜0.05 vs. DM; ^$^P＜0.05 vs. NE; ^&^P＜0.05 vs. ACh; ^#^P＜0.05 vs. ACh+NE.

**sFigure.18. ACh involved in sarcomere and metabolic-related genes expression**

(A-D) ACh involved in sarcomere and metabolic-related genes expressions as determined by western blot (A) and semi-quantitative analysis (B-D). n=3,*P＜0.05 vs. Ctrl; ^&^P＜0.05 vs. 10^-5^ Mol/L ACh; ^#^P＜0.05 vs. 10^-5^ Mol/L ACh.

**sFigure.19. ACh reversed the effects of NE on C2C12 cells differentiation and muscle fibers types through FOXO3A signaling**

1. ACh improved myotube formation of C2C12 myoblasts cells inhibited by NE under differentiation medium(DM), and the specific effects could be obviously abolished by over-expressing FOXO3A as determined by MyHC staining. (B)C2C12 myoblasts cells transfected with Ad-FOXO3A (MOI:100) under differentiation medium (DM) with continuous 10-5 Mol/L NE and /or 10-8 Mol/L ACh for 6 days were determined by MyHC staining. n=9, *P＜0.05 vs. DM; ^#^P＜0.05 vs. DM; ^@^P＜0.05 vs. NE; ^&^P＜0.05 vs. ACh;^$^P＜0.05 vs. NE+ACh. (C-F) ACh reversed the effects of NE on C2C12 cells differentiation and muscle fibers types as determined by real-time PCR. n=3, *P＜0.05 vs. DM; ^#^P＜0.05 vs. DM; ^@^P＜0.05 vs. NE; ^&^P＜0.05 vs. ACh;^$^P＜0.05 vs. NE+ACh.

**sFigure.20. The hearts with VNS treatment were immunostained with normal IgG (negative control).**

The hearts with VNS treatment were immunostained with normal IgG (negative control). (A) Typical image of negative staining by HRP. The section was counterstained with haematoxylin. (B) Typical image of negative staining by TRITC (Red). DAPI was used to stain the cell nuclei (blue).
