# Supplementary figures and images for: Vagus nerve stimulation optimized cardiomyocyte phenotype, sarcomere organization and energy metabolism in infarcted heart through FoxO3A-VEGF signaling
Source: Cell Death Dis. 2020 Nov 12;11(11):971. doi: 10.1038/s41419-020-03142-0 (PMC7665220; doi:10.1038/s41419-020-03142-0)

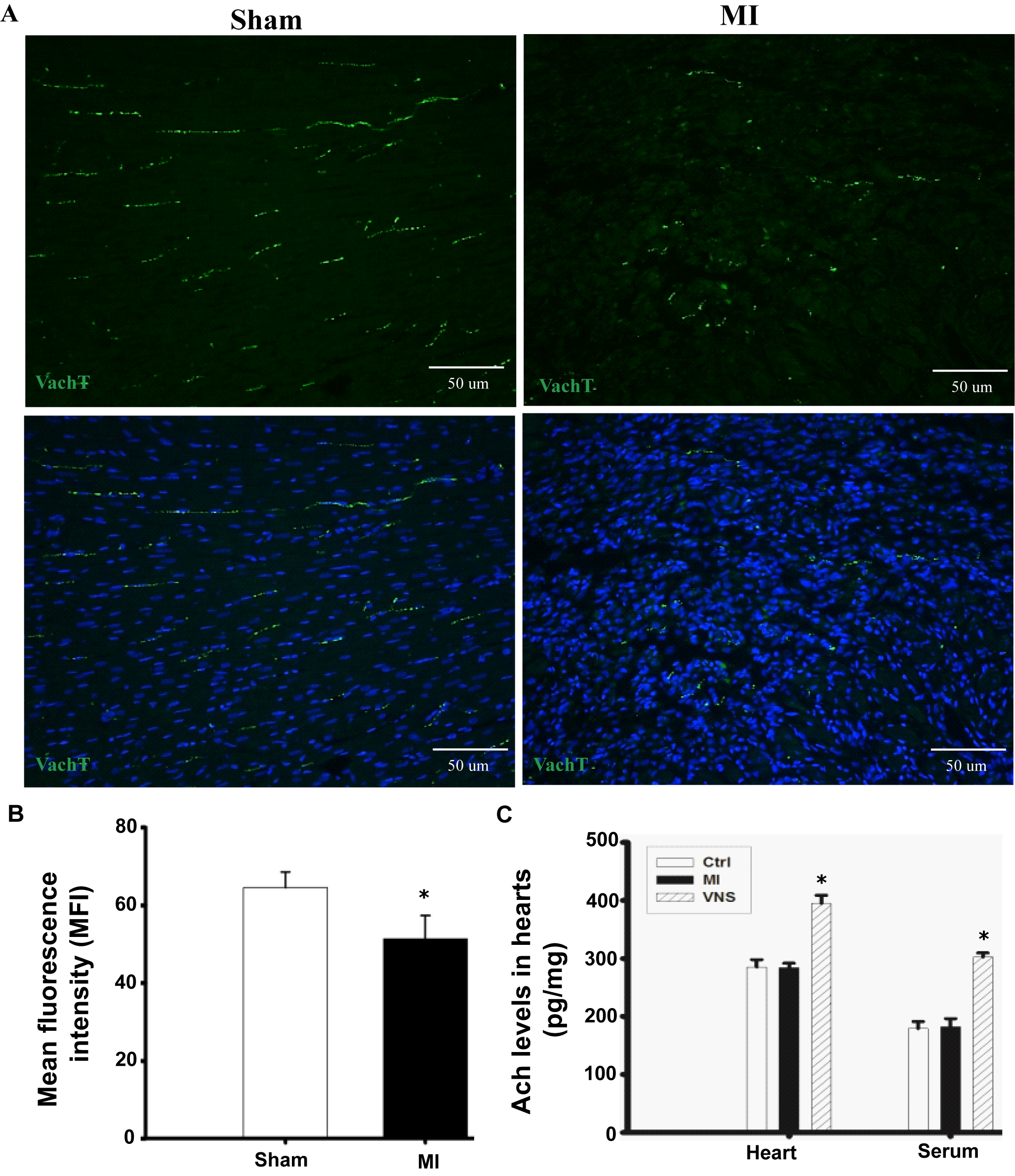

Supplement: Supplementary file 2 — FIgure 1 [file 41419_2020_3142_MOESM2_ESM.tif]

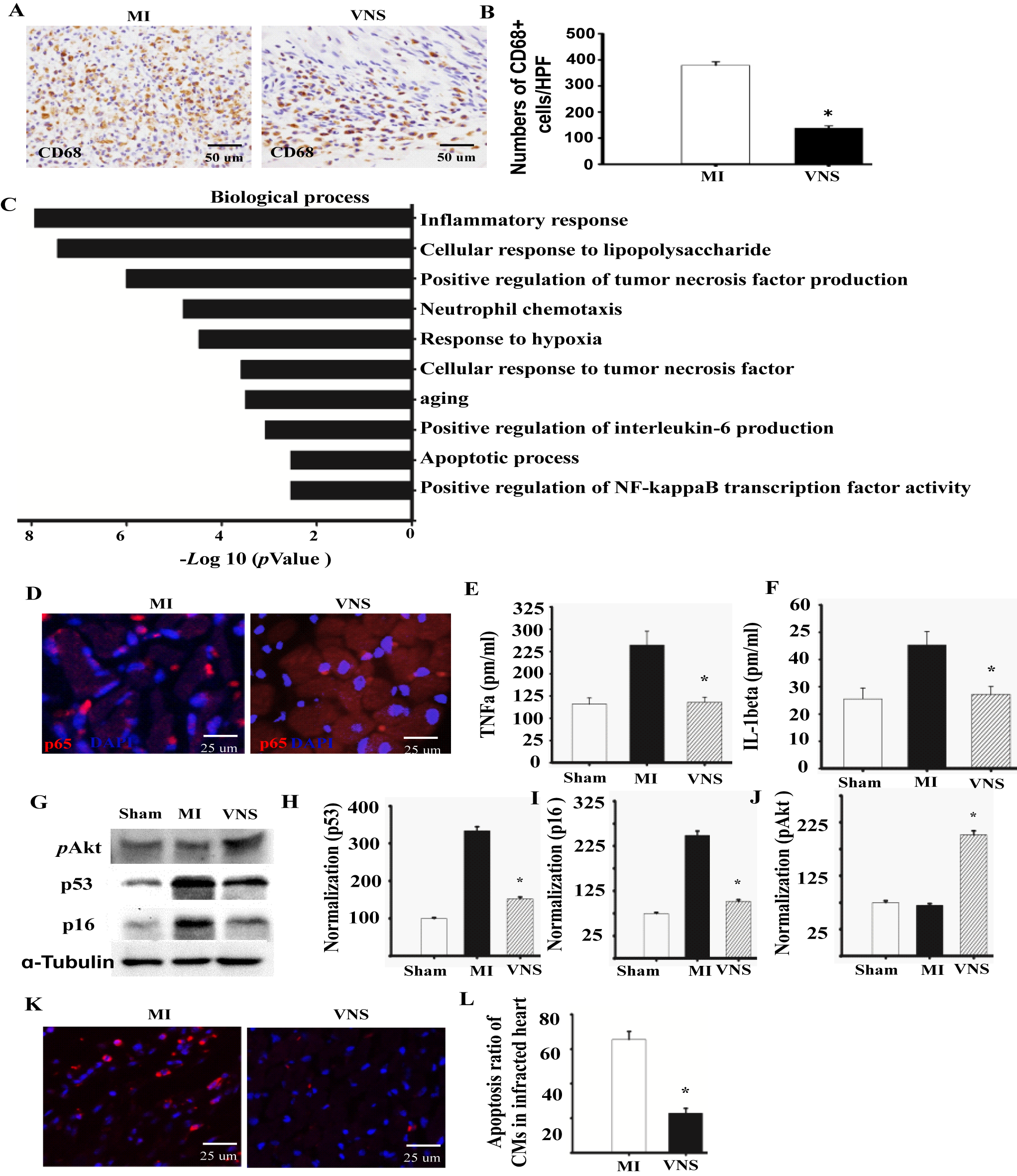

Supplement: Supplementary file 3 — Figure 2 [file 41419_2020_3142_MOESM3_ESM.tif]

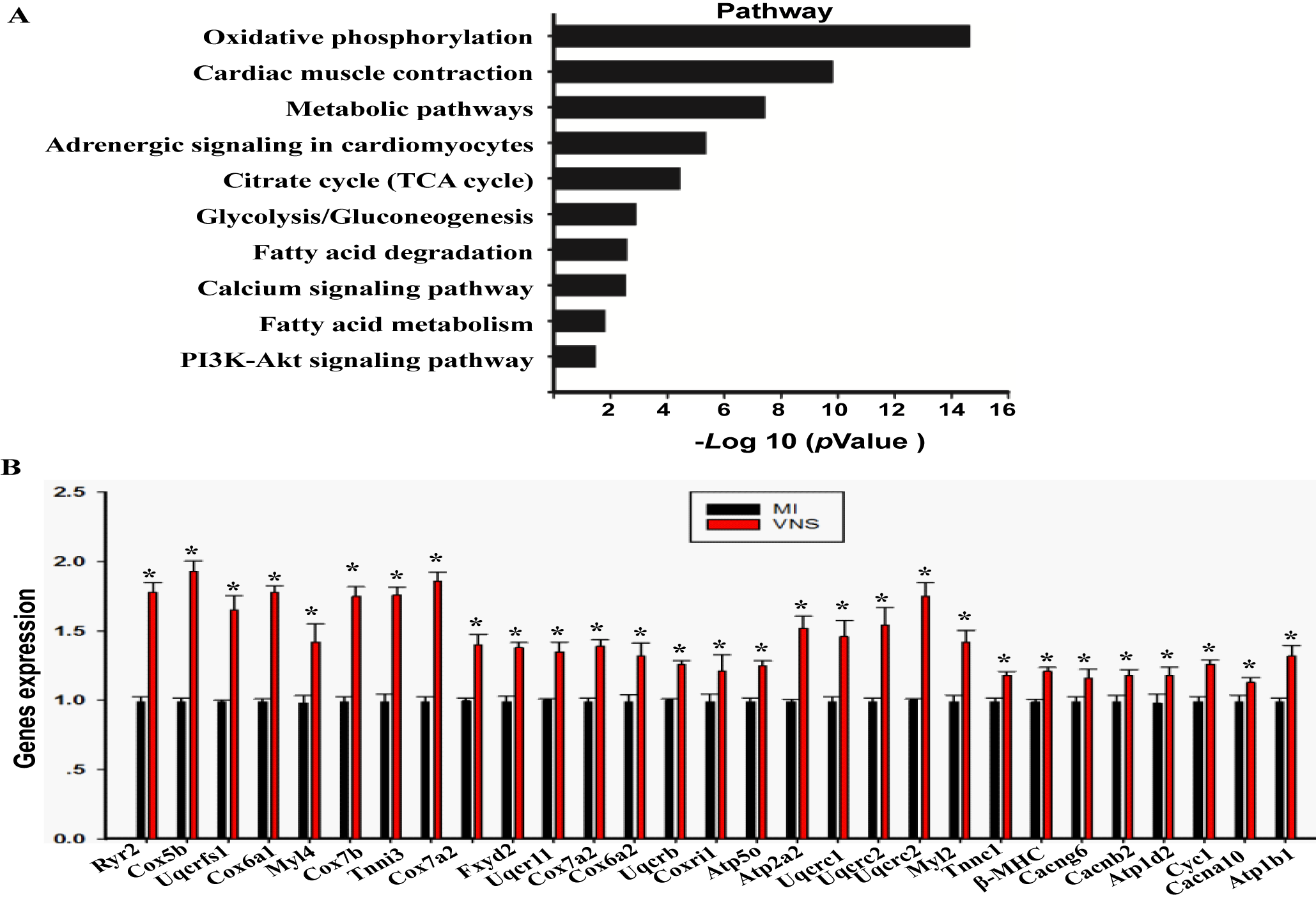

Supplement: Supplementary file 4 — Figure 3 [file 41419_2020_3142_MOESM4_ESM.tif]

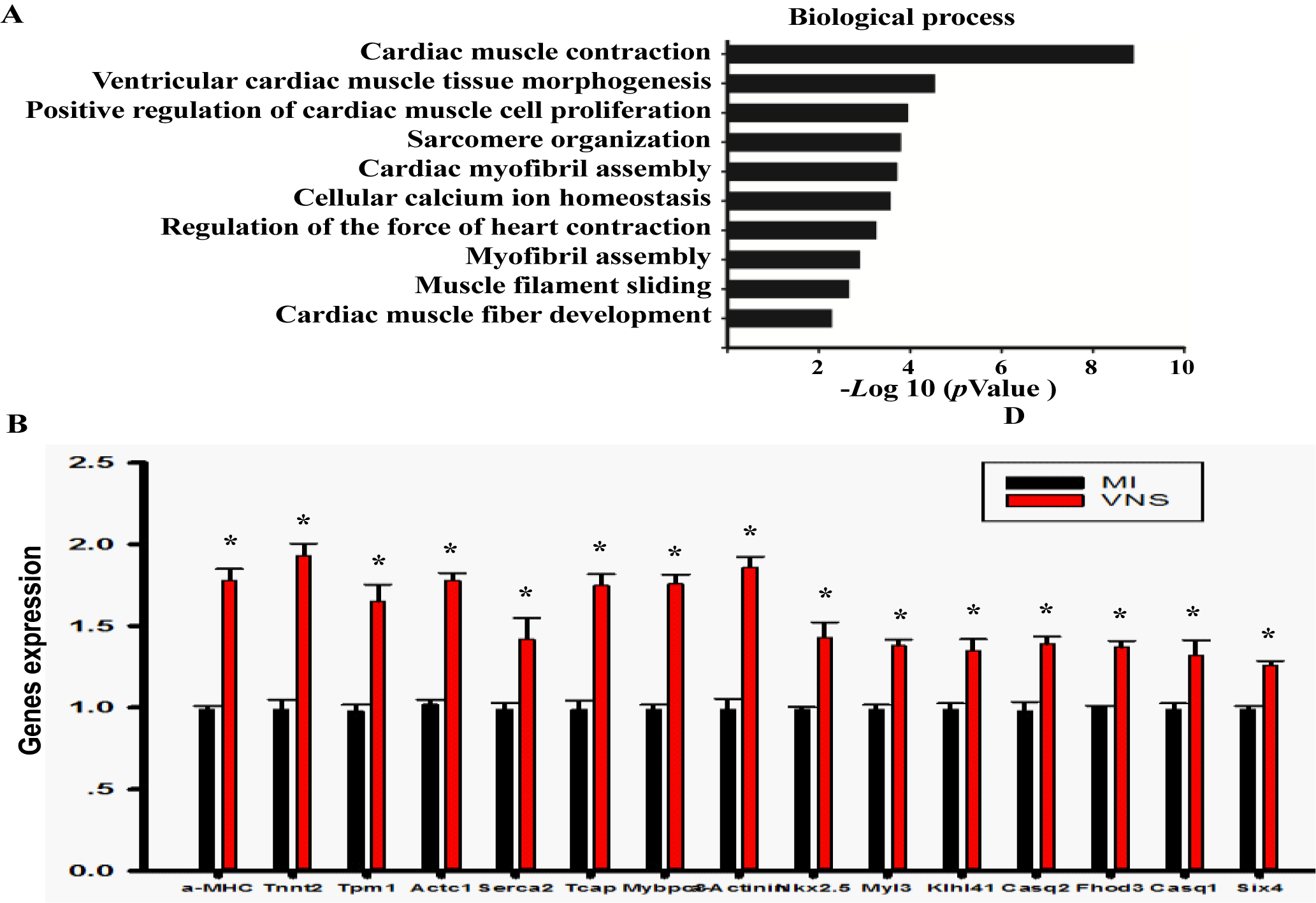

Supplement: Supplementary file 5 — Figure 4 [file 41419_2020_3142_MOESM5_ESM.tif]

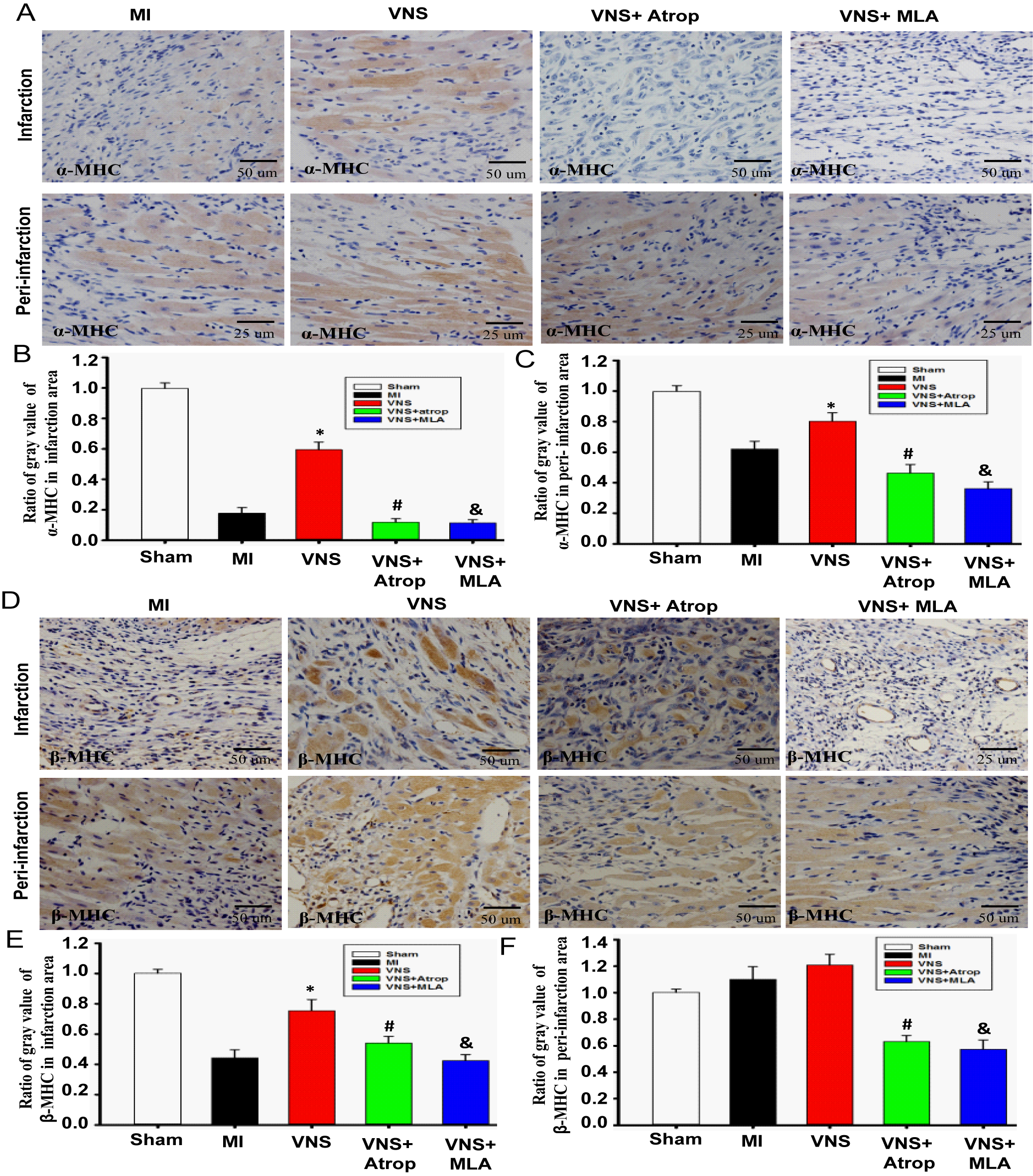

Supplement: Supplementary file 6 — Figure 5 [file 41419_2020_3142_MOESM6_ESM.tif]

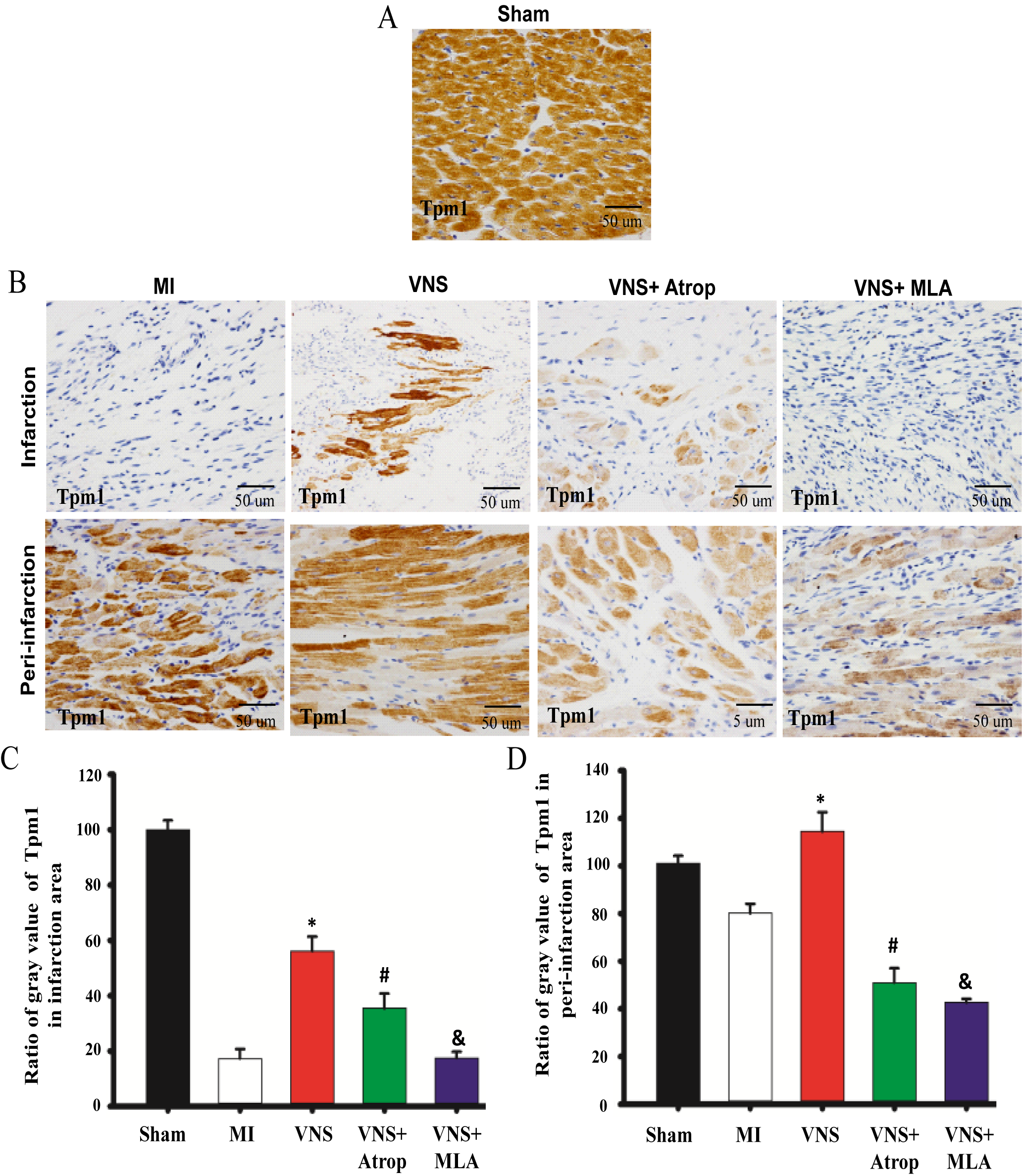

Supplement: Supplementary file 7 — Figure 6 [file 41419_2020_3142_MOESM7_ESM.tif]

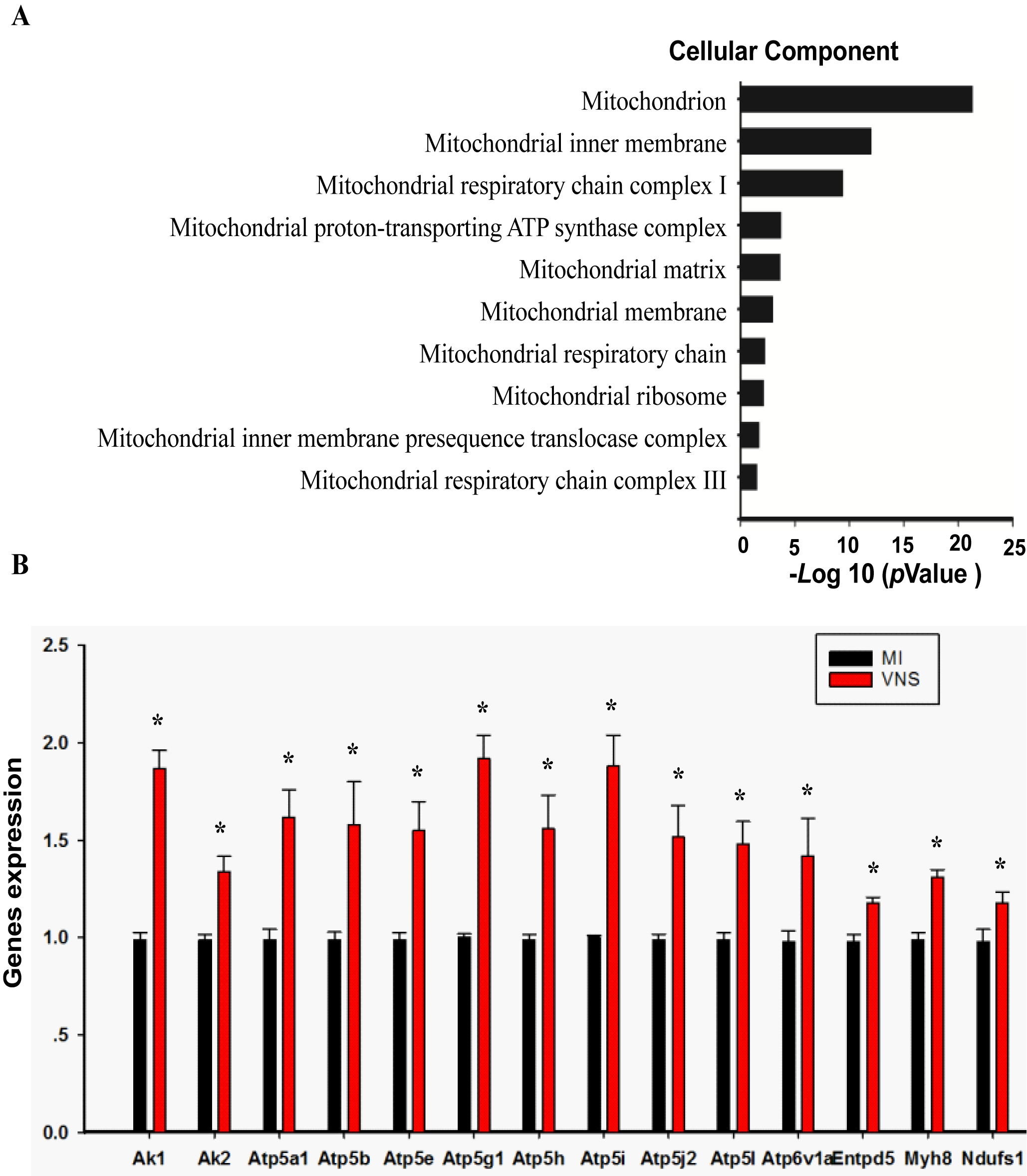

Supplement: Supplementary file 8 — Figure 7 [file 41419_2020_3142_MOESM8_ESM.tif]

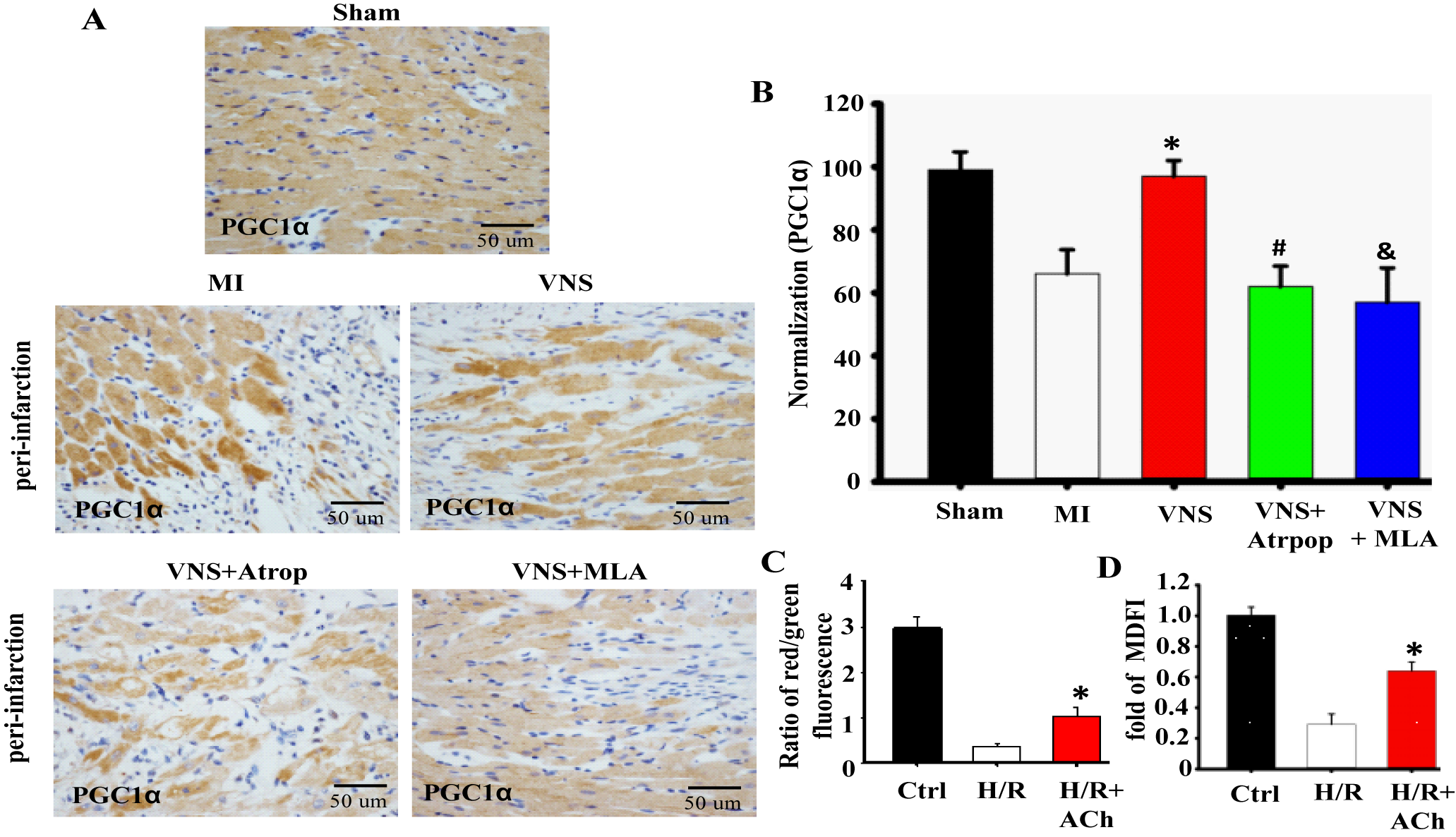

Supplement: Supplementary file 9 — Figure 8 [file 41419_2020_3142_MOESM9_ESM.tif]

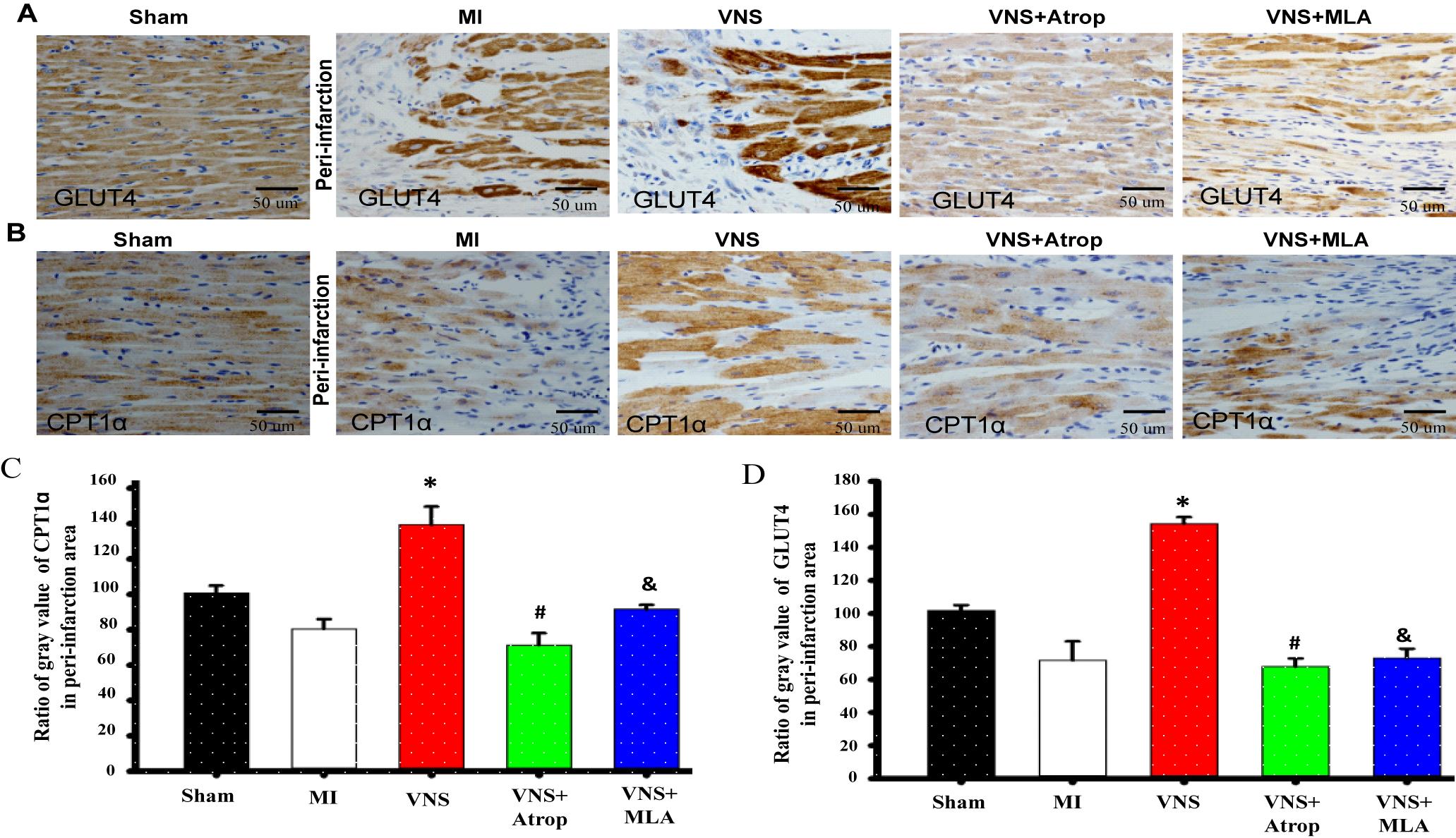

Supplement: Supplementary file 10 — Figure 9 [file 41419_2020_3142_MOESM10_ESM.tif]

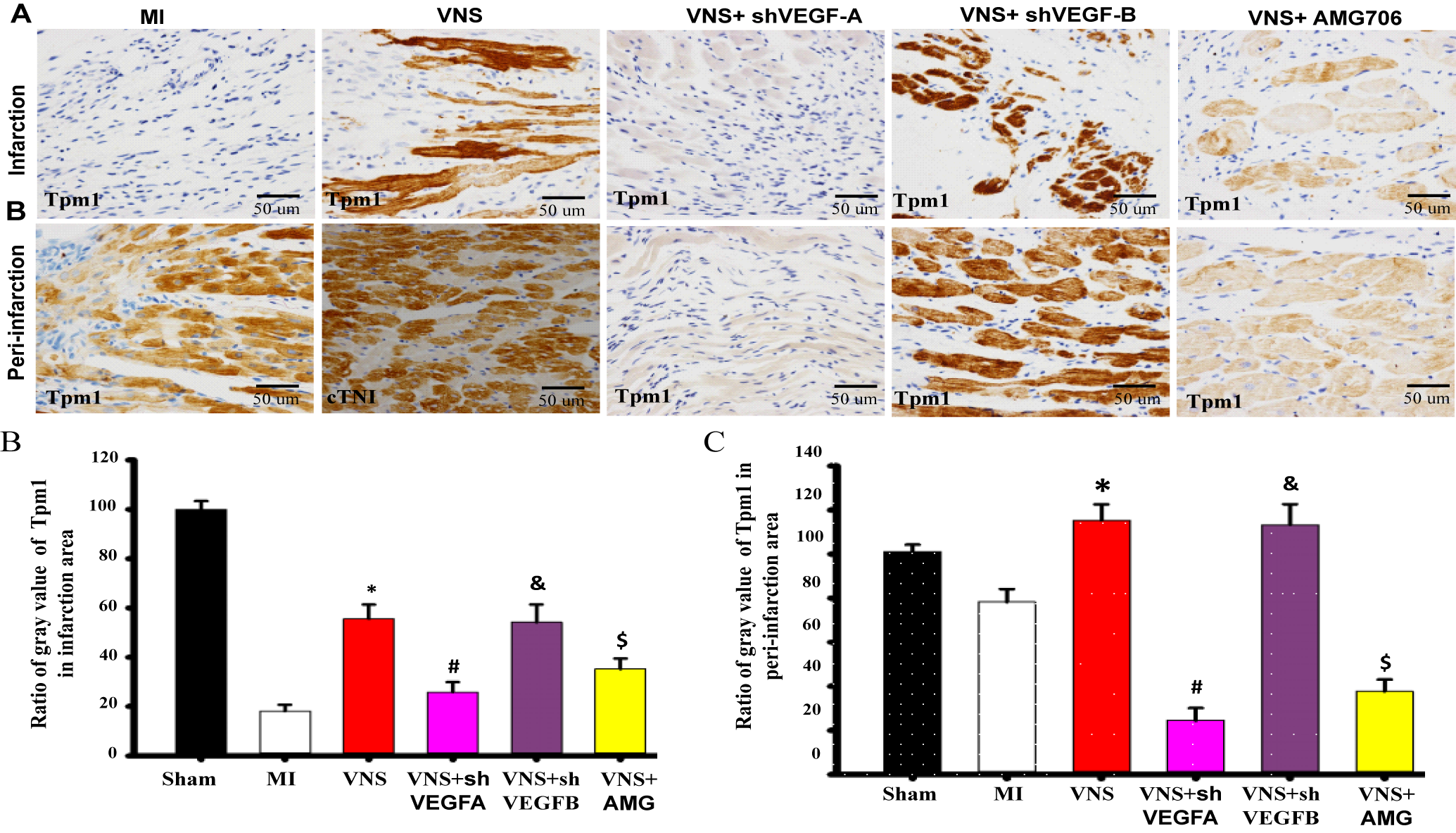

Supplement: Supplementary file 11 — Figure 10 [file 41419_2020_3142_MOESM11_ESM.tif]

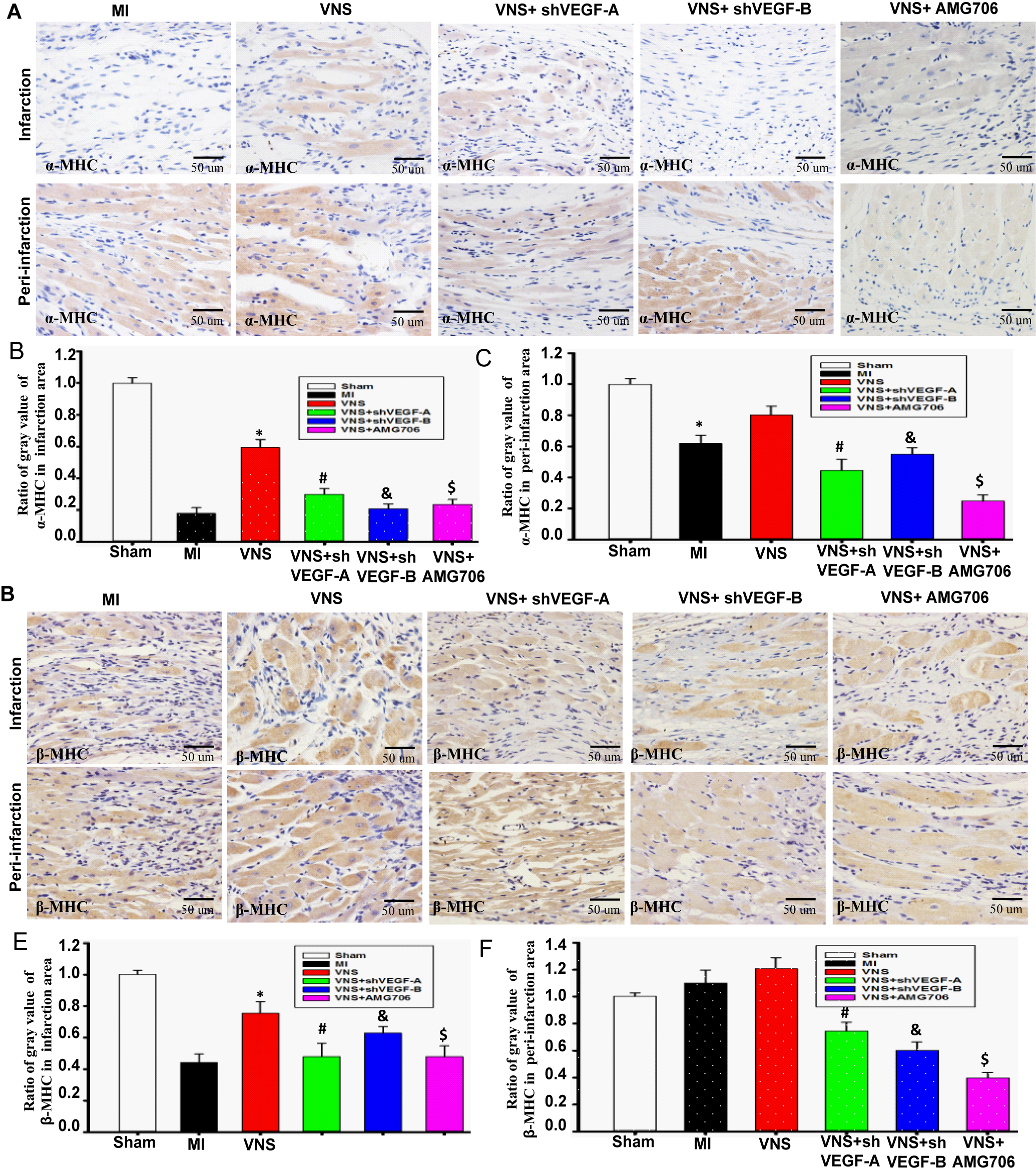

Supplement: Supplementary file 12 — Figure 11 [file 41419_2020_3142_MOESM12_ESM.tif]

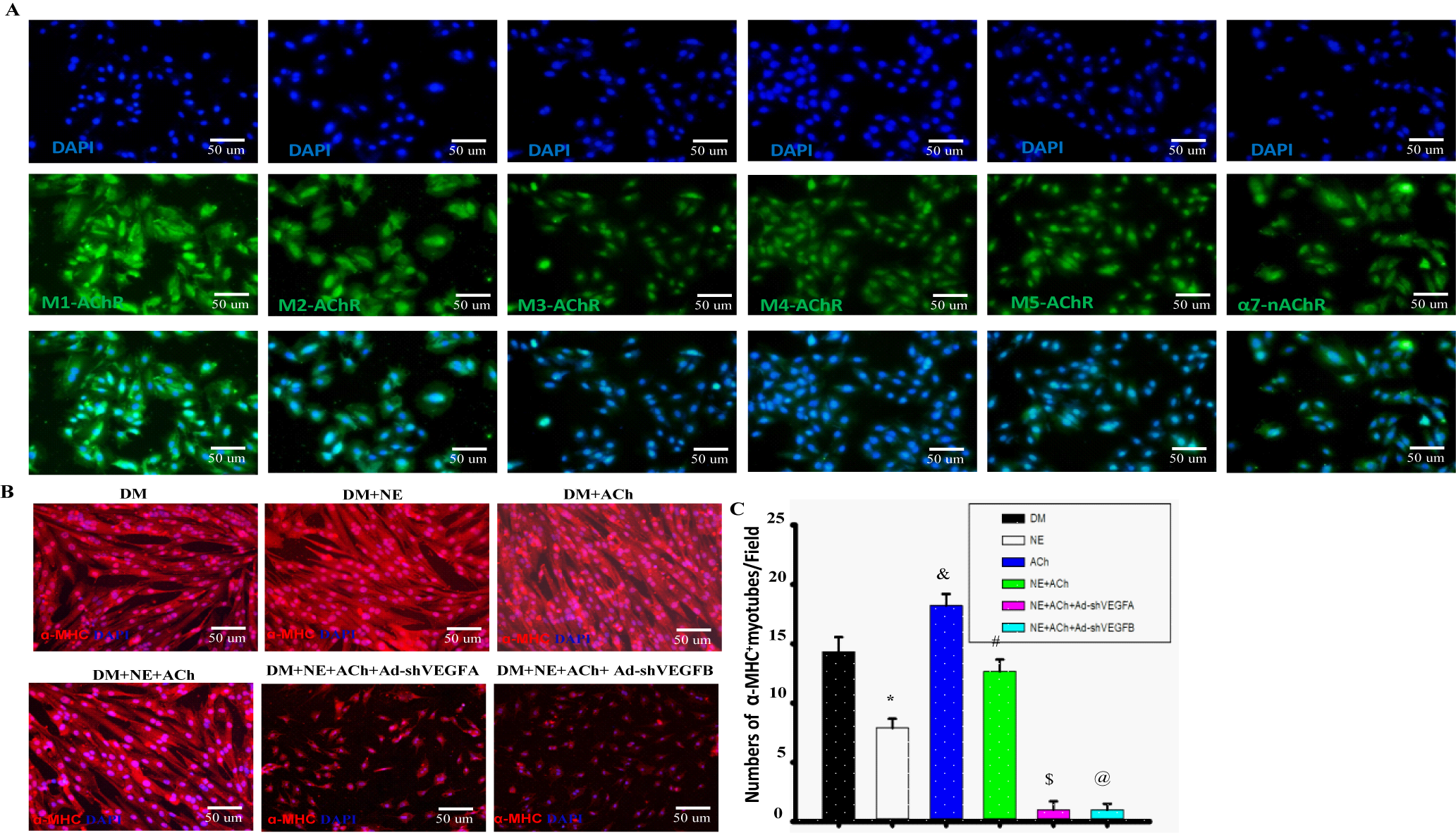

Supplement: Supplementary file 13 — Figure 12 [file 41419_2020_3142_MOESM13_ESM.tif]

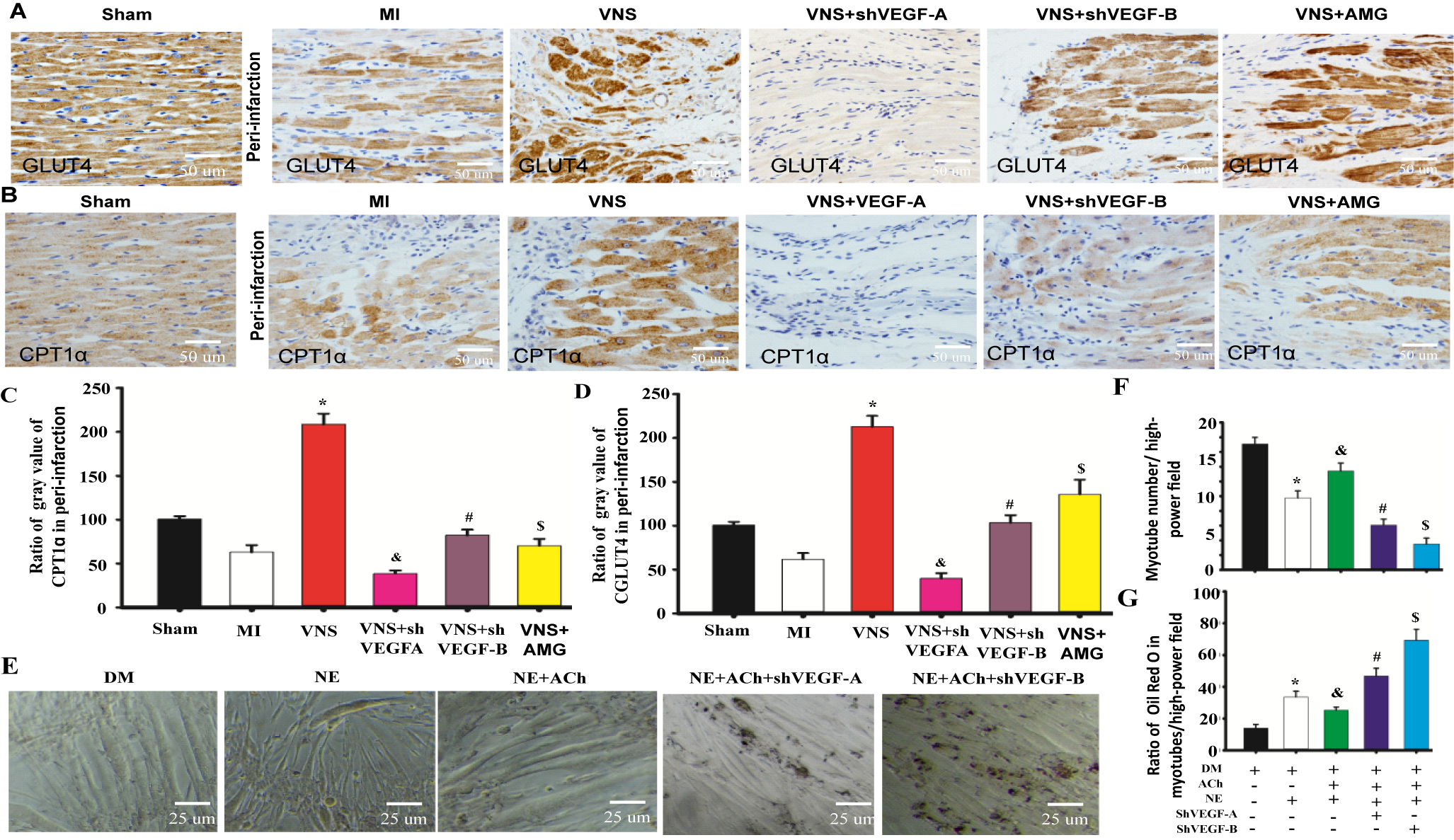

Supplement: Supplementary file 14 — Figure 13 [file 41419_2020_3142_MOESM14_ESM.tif]

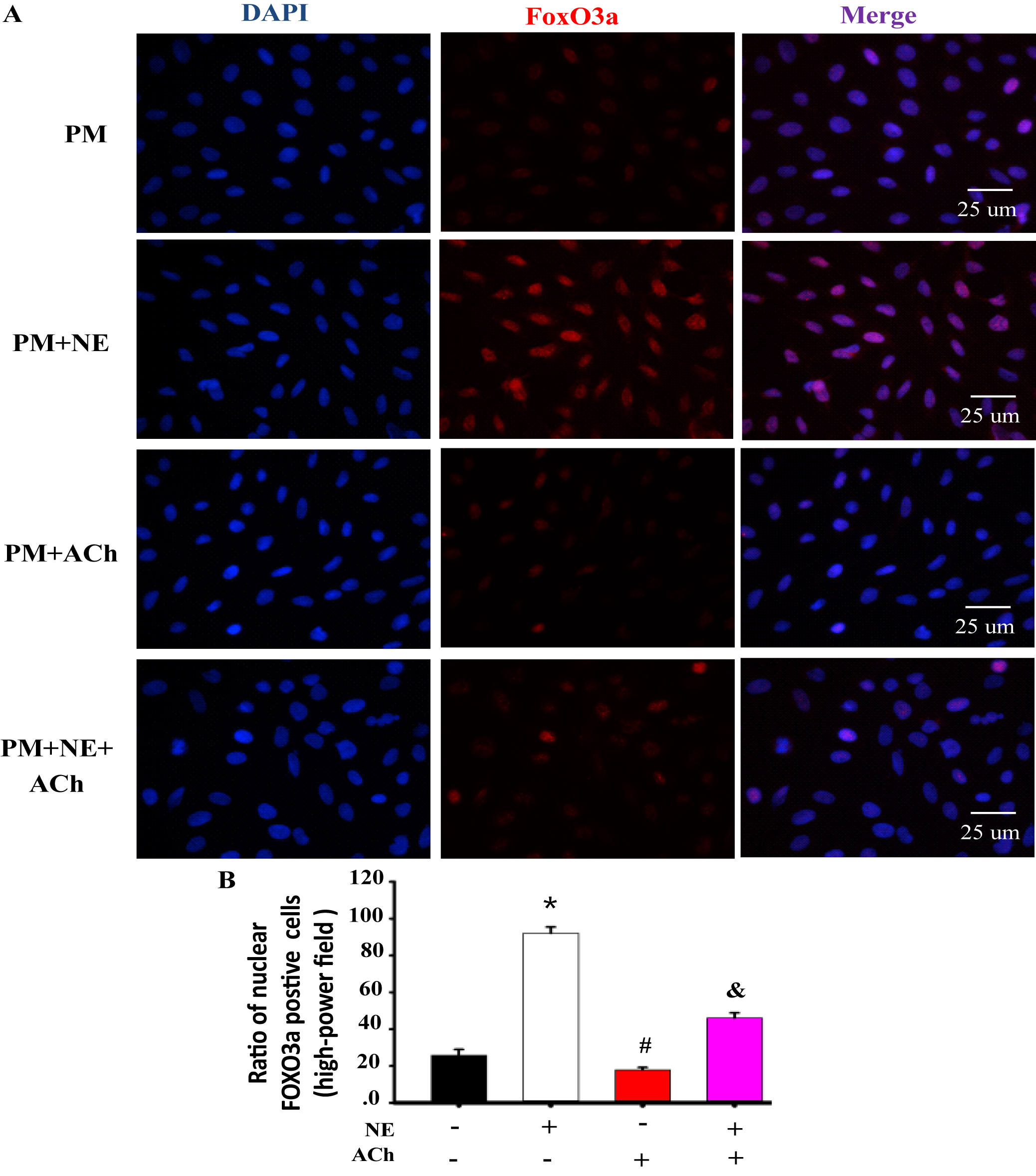

Supplement: Supplementary file 15 — Figure 14 [file 41419_2020_3142_MOESM15_ESM.tif]

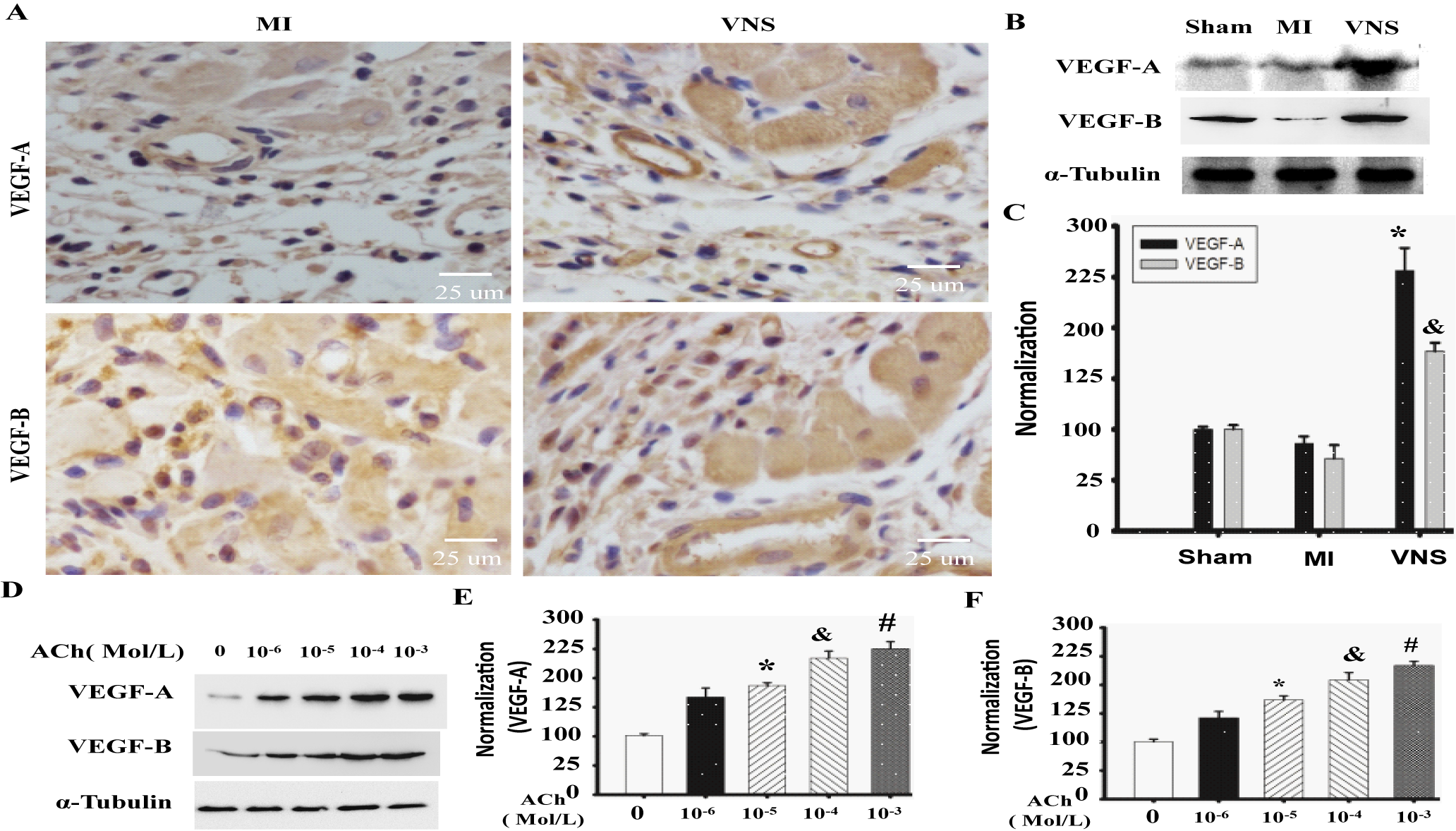

Supplement: Supplementary file 16 — Figure 15 [file 41419_2020_3142_MOESM16_ESM.tif]

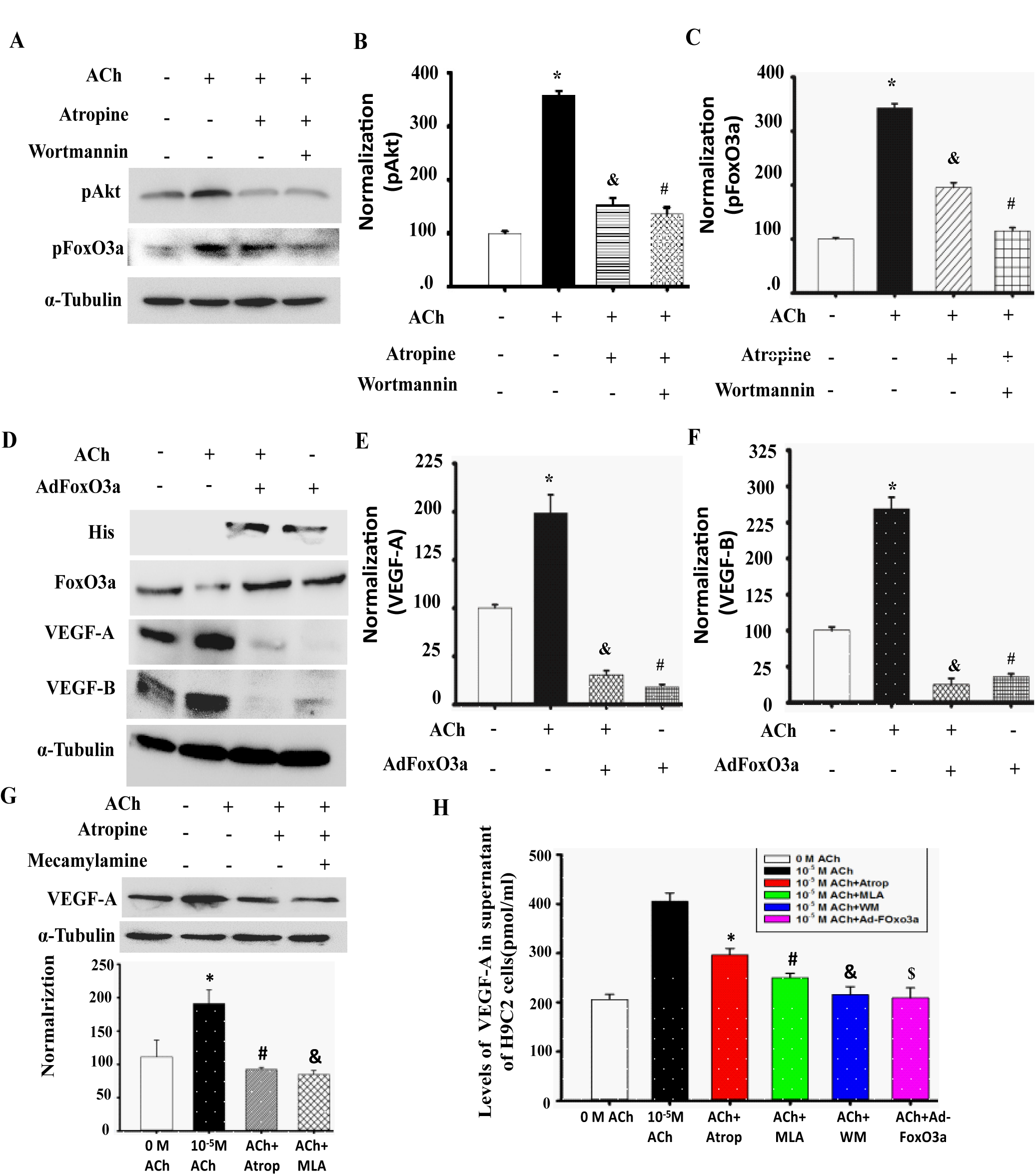

Supplement: Supplementary file 17 — Figure 16 [file 41419_2020_3142_MOESM17_ESM.tif]

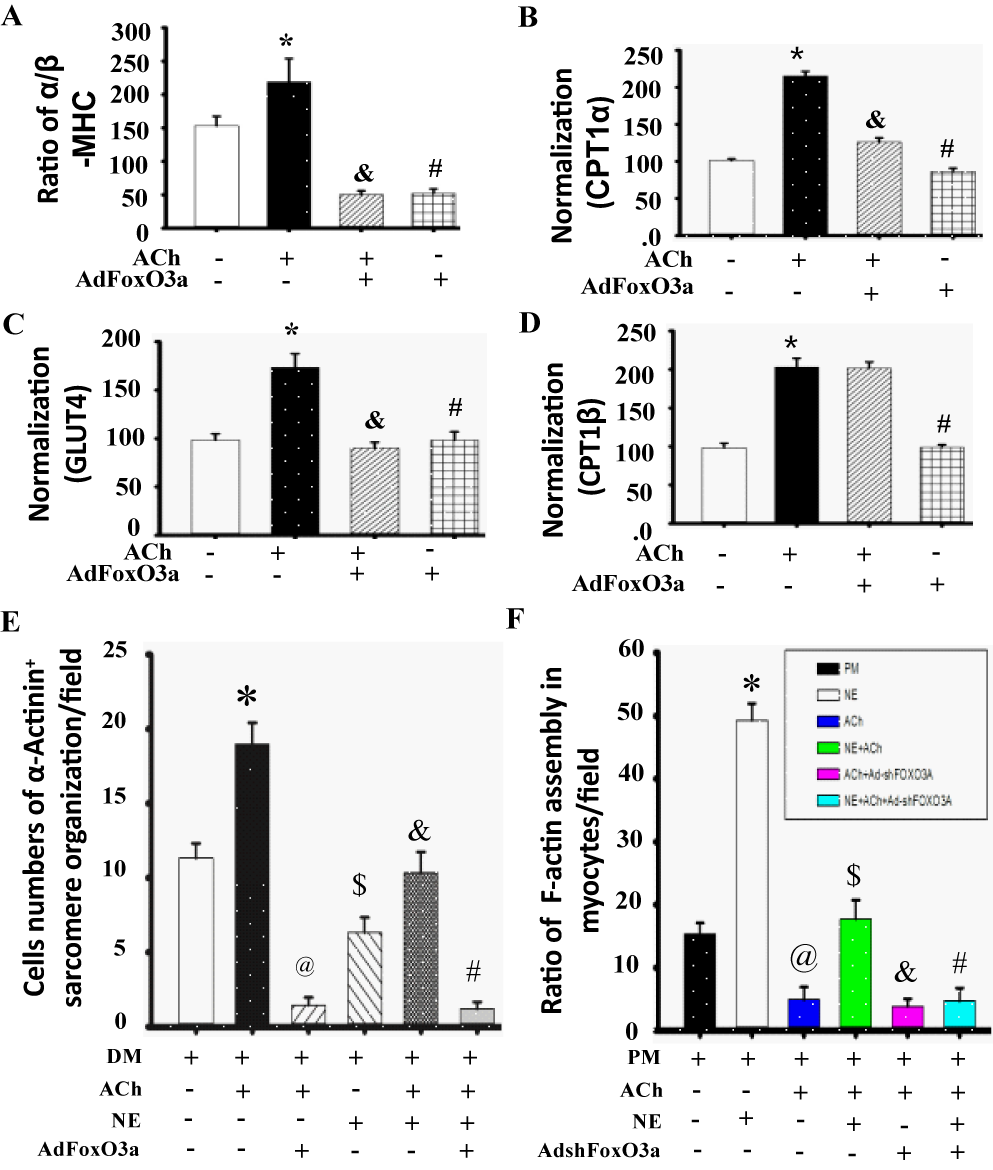

Supplement: Supplementary file 18 — Figure 17 [file 41419_2020_3142_MOESM18_ESM.tif]

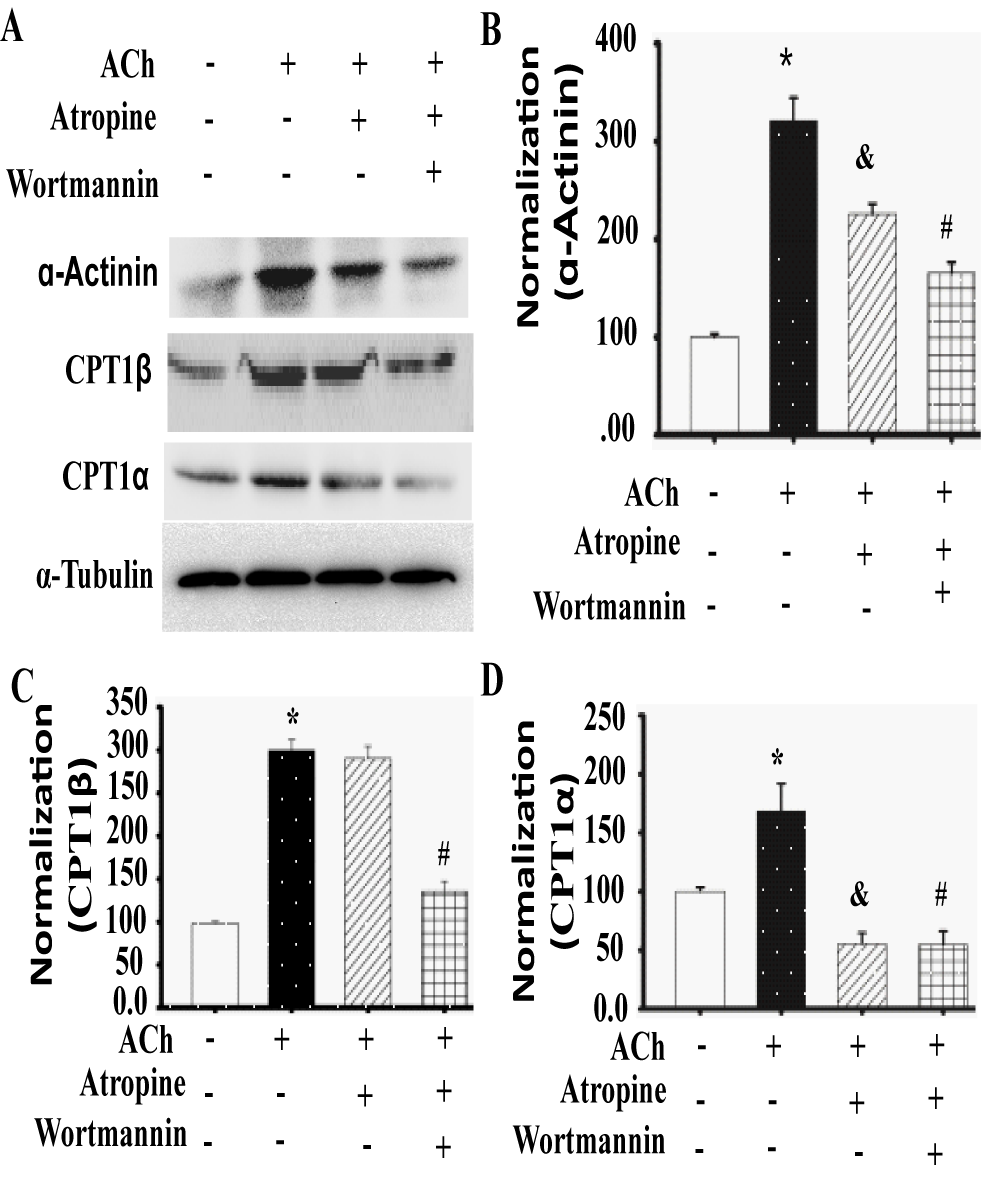

Supplement: Supplementary file 19 — Figure 18 [file 41419_2020_3142_MOESM19_ESM.tif]

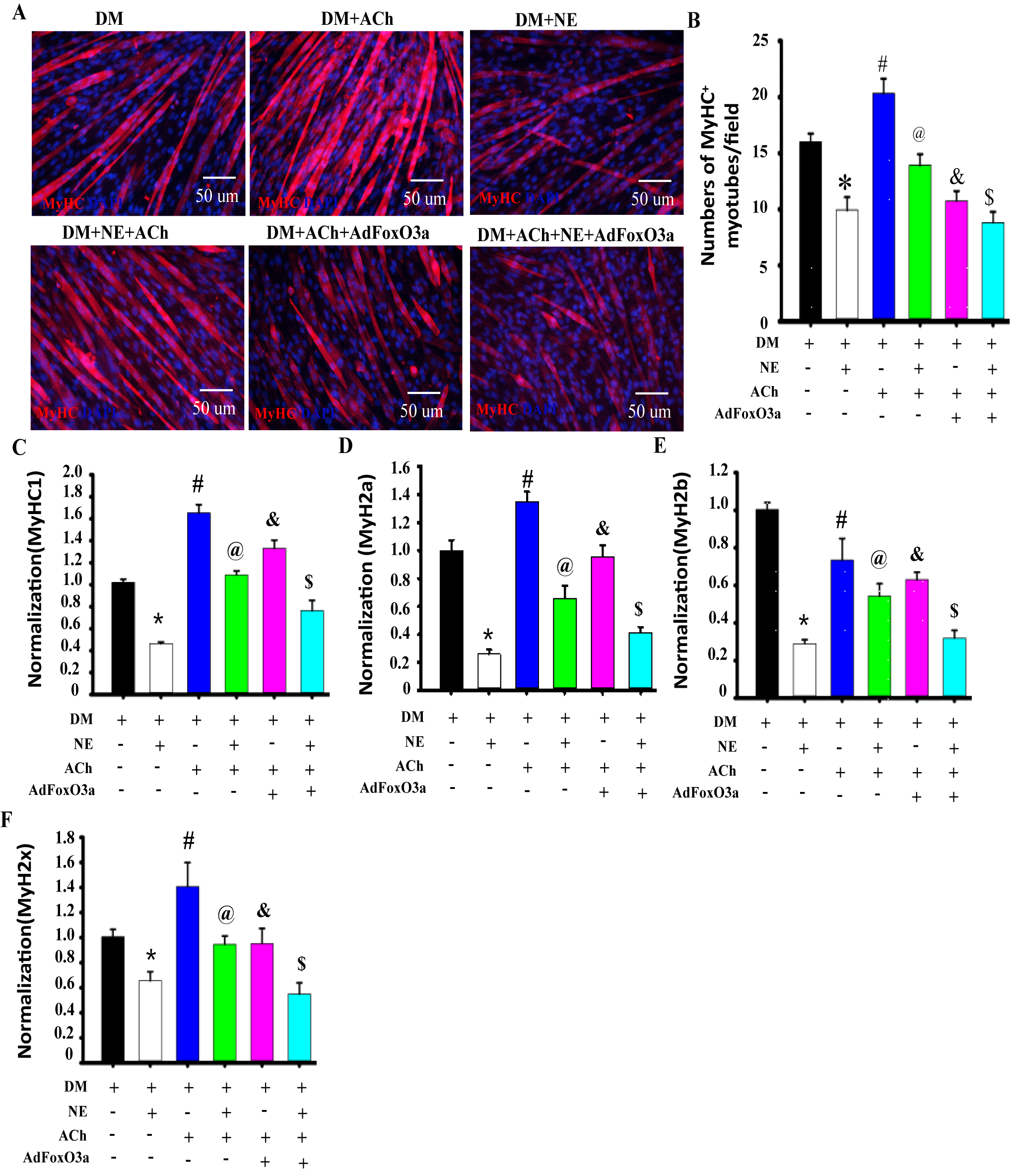

Supplement: Supplementary file 20 — Figure 19 [file 41419_2020_3142_MOESM20_ESM.tif]

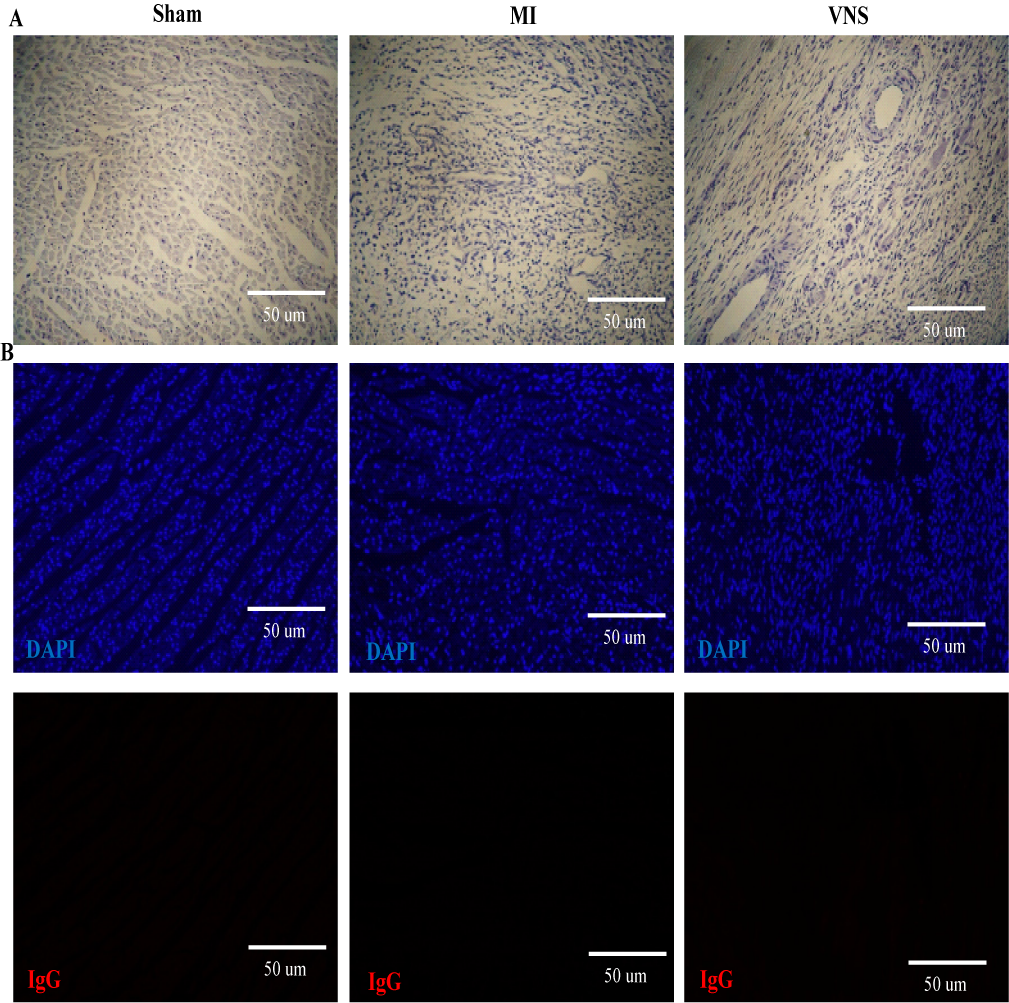

Supplement: Supplementary file 21 — Figure 20 [file 41419_2020_3142_MOESM21_ESM.tif]
